# Total Synthesis and Antifungal Activity of Palmarumycin CP_17_ and Its Methoxy Analogues

**DOI:** 10.3390/molecules21050600

**Published:** 2016-05-07

**Authors:** Ruina Wang, Guoyue Liu, Mingyan Yang, Mingan Wang, Ligang Zhou

**Affiliations:** 1 Department of Applied Chemistry, China Agricultural University, Beijing 100193, China; wrn0722@sina.com (R.W.); liuguoyue001@126.com (G.L.); yangmy@cau.edu.cn (M.Y.); 2 Department of Plant Pathology, China Agricultural University, Beijing 100193, China

**Keywords:** spirobisnaphthalene, palmarumycin CP_17_, total synthesis, ketalization, antifungal activities

## Abstract

Total synthesis of naturally occurring spirobisnaphthalene palmarumycin CP_17_ and its methoxy analogues was first achieved through Friedel-Crafts acylation, Wolff-Kishner reduction, intramolecular cyclization, ketalization, benzylic oxidation, and demethylation using the inexpensive and readily available methoxybenzene, 1,2-dimethoxybenzene and 1,4-dimethoxybenzene and 1,8-dihydroxynaphthalene as raw materials. Demethylation with (CH_3_)_3_SiI at ambient temperature resulted in ring A aromatization and acetal cleavage to give rise to binaphthyl ethers. The antifungal activities of these spirobisnaphthalene derivatives were evaluated, and the results revealed that **5** and **9b** exhibit EC_50_ values of 9.34 µg/mL and 12.35 µg/mL, respectively, against *P. piricola*.

## 1. Introduction

**Scheme 1:**
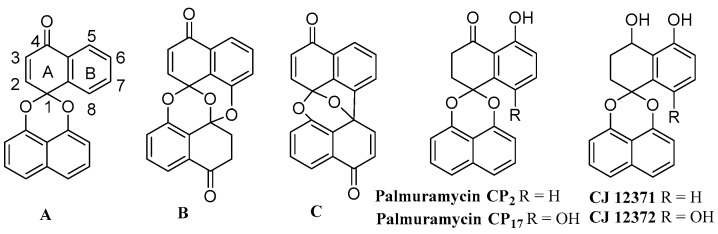
Three types of basic spirobisnaphthalene structures (A–C), palmarumycin CPs and CJs.

The spirobisnaphthalenes are a group of natural products, possessing a bisnaphthoquinone spiroketal moiety as a common structural feature, which display a variety of biological activities such as antibacterial, antifungal, antileishmanial, enzyme-inhibitory and antitumor activities [1]. The spirobisnaphthalenes have been classified into three subtypes based on their number of spiroketal units (Scheme 1). The most typical subtype, Type A, has a spiroketal structure composed of a 1,8-dihydroxy naphthalene and a naphthoquinone. In Type B, two naphthyl structural units are connected with an additional ether linkage to the core bisnaphthoquinone spiroketal structure. Finally, Type C has a binaphthyl moiety in place of the Type B′s binaphthyl ether structure [1]. Recently, a new spirobisnaphthalene subclass was isolated from the saprobic fungus *Urnula craterium* [2]. These are structurally distinct in that they contain a 1,8-dihydroxynaphthalene–derived spiroether unit bridged through a spiroether linkage. The spirobisnaphthalenes have attracted significant attention not only because of their unique structures, but also because they display a range of biological activities. In 1997, Wipf and Jung reported the first synthetic study toward diepoxin σ; their strategy has become a typical approach to synthesize this class of natural products [3]. A comprehensive review outlining synthetic approaches to these metabolites was published in 2005 [1]. Since then, several new spirobisnaphthalene natural products have been isolated and the total syntheses of some of these have been accomplished by different research groups. In addition, many non-natural derivatives have been prepared by synthesis and evaluated for their biological activities [4,5,6,7,8,9,10,11,12,13,14,15,16,17,18]. The spirobisnaphthalenes isolated from fungi along with their biological activities were further reviewed in 2010 [19,20]. We have explored the identification of spirobisnaphthalenes from the endophytic fungus *Berkleasmium* sp. Dzf12, which was an isolate from the medicinal plant *Dioscorea zingiberensis.* It followed that a variety of bioactive spirobisnaphthalenes and the preparative separation of spirobisnaphthalenes could be separated on a preparative basis by high-speed counter-current chromatography from extracts of *Berkleasmium* sp. Dzf12 [21,22,23]. It emerged also that the production of some of these metabolites could be enhanced in liquid culture by the addition of oligosaccharides, polysaccharides, or yeast extract, and that they could be most efficiently isolated by resin adsorption.

A racemic synthesis of the spiroxin framework using a desymmetrization approach has been reported [24]. This was followed by a reported catalytic asymmetric approach to construct the tertiary naphthoquinol stereogenic center present in the spiroxin framework [25]. Since the first synthesis of racemic spiroxin C and several preussomerin analogues, there have been no new syntheses of Type B and C spirobisnaphthalenes since 2004, due to their synthetic complexity [19,20,24], so attention remained focused on elaborations of the type A class and their bioactivity evaluation [16,17,18].

**Scheme 2:**
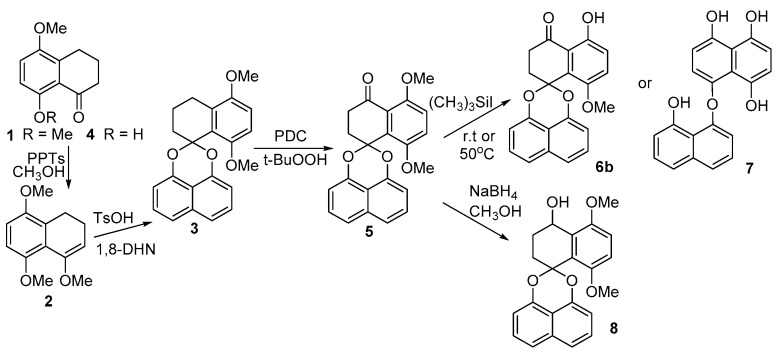
The synthesis of intermediates **3**, **5**, 8-methoxy analogue **6b**, binaphthyl ether **7** and **5**, 8-dimethoxy CJ 12372 **8**.

**Scheme 3:**
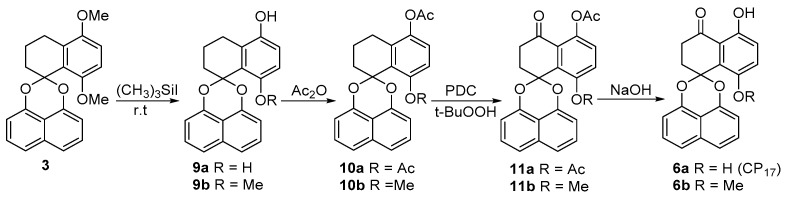
The synthesis of palmarumycin CP_17_ **6a** and 8-methoxy analogue **6b**.

**Scheme 4:**
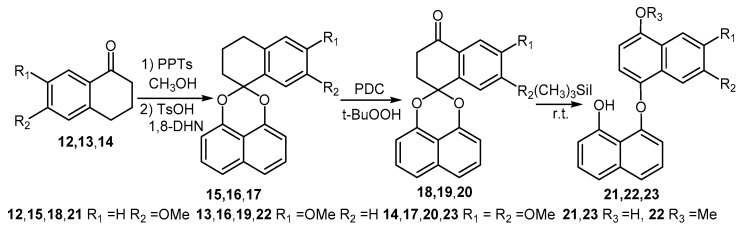
Synthesis of palmarumycin CP_17_ analogues **18**–**20** and demethylation products of **5**, **18** and **19**.

Palmarumycin CP_17_ is a new type A spirobisnaphthalene isolated from the Panamanian endophytic fungus *Edenia* sp and the endophytic fungus Dzf12 of *Dioscorea zingiberensis*. It has strong antileishmanial and antimicrobial activities [26,27,28]. Total syntheses of type A spirobisnaphthalenes have been achieved via four different approaches based on the different construction protocols of the core spiroketal structure. These include a biomimetic synthesis by oxidative cyclization to the binaphthyl ether [29,30], direct acetalization [31,32], a silver-mediated cationic cyclization following Suzuki-Miyamura cross-coupling [33], and a Diels-Alder approach in combination with the biomimetic oxidative cyclization of naphthyl phenyl ether [16]. Although the total syntheses of a range of similar palmarumycins, including CP_1_, CP_2_ and CJ-12371, was accomplished by direct acetalization as the key step [31,32,33,34,35], the existence of the sensitive 8-hydroxyl or 8-chlorine substituents found in type A spirobisnaphthalenes such as CJ 12372, ascochytain, palmarumycin B_6_, CP_17_, and CP_18_ offer a new challenge. In order to gain insights into the structure-activity relationships of both natural and non-natural spirobisnaphthalenes, we have used the direct acetalization approach, following the synthetic protocol of preussomerin G and I [31,32,36], to complete the total synthesis of palmarumycin CP_17_ (**6a**), its 8-methoxy analogue (**6b**), and 5,8-dimethoxy CJ 12372 (**8**) as well as the other 6-methoxy,7-methoxy and 6,7-dimethoxy spirobisnaphthalene derivatives (**18**–**20**) (Scheme 2, Scheme 3 and Scheme 4). These compounds were then evaluated for their antifungal activities. Here we present our results.

## 2. Results and Discussion

### 2.1. Synthesis of Palmarumycin CP_17_ and Its Methoxy Analogues

Of the four main approaches to the construction of the core spiroketal structure, direct acetalization offers the most economical approach to palmarumycin CP_17_ due to the available starting materials. The presence of the C_8_ hydroxyl group renders the direct acetalization between tetralone **1** and 1,8-dihydroxynaphthalene (1,8-DHN) problematic and the spiroketal core **3** of palmarumycin CP_17_ was not accessible in this way. Similarly, the reaction between 5-methoxy-8-hydroxytetralone **4** and 1,8-DHN was unsuccessful. Tetralone **4** was obtained by the demethylation of 5,8-dimethoxy tetralone with (CH_3_)_3_SiI; however, the intramolecular hydrogen bonding is probably responsible for this failure. Nonetheless, we found that **1** could be easily transferred into enol ether **2** in high yield with pyridine *p*-toluenesulfonate (PPTs) as a catalyst and under an atmosphere of N_2_. Product **2** required isolation over neutral Al_2_O_3_ chromatography. The reaction of ketoenol ether **2** with 1,8-DHN in the presence of TsOH under a strict oxygen and moisture-free atmosphere readily afforded the key spirobisnaphthalene **3** (Scheme 2) [37]. The benzylic oxidation of **3** using pyridine dichromate (PDC) and *t*-BuOOH gave spirobisnaphthalene **5** successfully. Selective mono-demethylation of **5** with (CH_3_)_3_SiI under ambient condition generated only the 8-methoxy analogue (**6b**) of palmarumycin CP_17_ (73% yield), but not the desired palmarumycin CP_17_ (**6a**) (Scheme 2). However, demethylation of **5** with (CH_3_)_3_SiI at 50 °C unexpectedly resulted in ring A aromatization and acetal cleavage to afford binaphthyl ether **7**, which was the same as the case reported in the reference (Scheme 2) [31,32]. The NaBH_4_ reduction of **5** produced 5,8-dimethoxy CJ 12372 (**8**) in 97% yield (Scheme 2); however, demethylation of **8** with (CH_3_)_3_SiI and BBr_3_, in an attempt to prepare CJ 12372, proved unsuccessful.

Based on the above observations, an alternative reaction sequence was explored (Scheme 3). In the new sequence, compounds **9a** and **9b** could be readily obtained in high yields without ring A aromatization and acetal cleavage when demethylation of **3** was carried out with a different quantity of (CH_3_)_3_SiI (Scheme 3) [29,30]. After the acetylation to generate **10a** and **10b**, benzylic oxidation with pyridine dichromate (PDC) and *t*-BuOOH afforded ketones **11a** (69%) and **11b** (55%), respectively (Scheme 3). Finally, spirobisnaphthalenes **11a** and **11b** were deacetylated to give palmarumycin CP_17_ **6a** (91%) and 8-methoxy analogue **6b** (93%), respectively. After the accomplishment of **6a** and **6b**, the spirobisnaphthalenes **15**–**17** were prepared by 1,8-DHN coupling with tetralones **12**–**14**, following the procedure carried out for **3**, and the benzylic oxidation as before gave spirobisnaphthalenes **18**–**20** (Scheme 4). However, demethylation of spirobisnaphthalenes **18** and **19** with (CH_3_)_3_SiI, even at room temperature, resulted in ring A aromatization and acetal cleavage to give rise to binaphthyl ethers **21**–**23** (Scheme 4), while demethylation of **20** with (CH_3_)_3_SiI at room temperature gave a complex product mixture, which could not be satisfactorily purified. Based on these results, we deduce that the hydroxyl and carbonyl groups at C_4_, the locations and numbers of the methoxyl groups on ring B and the reaction temperature are three factors which influence ring A aromatization and acetal cleavage to generate binaphthyl ethers. Nonetheless, with **5**, **10a** and **10b**, and **11a** and **11b** in hand, we have demonstrated a convenient method which should be amenable to the preparation of the other more complex natural products with a similar structure skeleton. Such targets are in progress in our laboratory.

### 2.2. Antifungal Activity of Spirobisnaphthalene Palmarumycin CP_17_ and Its Methoxy Analogues

The antifungal activity of the prepared compounds was evaluated using the mycelial growth rate test and the results are listed in Table 1 and Table 2. The data shows that these spirobisnaphthalenes exhibit higher inhibition against *P. piricola*, *R. solani*, *B. cinerea* and *C. arachidicolahori* in comparison to *F. oxysporum*, *F. graminearum* and *P. asaragisace*. The data in Table 1 illustrated that the hydroxyl and carbonyl groups at C_4_ play a crucial role for antifungal activity, while the demethylated or deactylated products exhibit much weaker activity than those with methoxy or acetyl groups. Palmarumycin CP_17_ **6a** and 8-methoxyl analogue **6b** had very similar inhibition potency against all seven phytopathogens, which indicates the hydroxyl/methoxyl groups at C_8_ have no significant impact on the antifungal activities. The inhibitory rates for **5** and **9b** against *P. piricola* were 73.7% and 75.0%, respectively, and the inhibition rate of **11b** against *R. solani* was 71.3%. In addition, the EC_50_ values of **5**, **9b** and **11b** were further determined to be 9.34, 12.35 and 11.18 µg/mL, respectively, and illustrated further the significant antifungal activities of these phytopathogens.

## 3. Materials and Methods

### 3.1. General Information

All reactions were performed under an N_2_ atmosphere. Unless otherwise stated, all reagents were purchased from commercial suppliers and used without further purification. Organic solvents were concentrated under reduced pressure using a rotary evaporator or oil pump. Flash column chromatography was performed using Qingdao Haiyang flash silica gel (200–300 mesh). Meting points were measured on a Yanagimoto apparatus and uncorrected. Infrared spectra were recorded using a Shimadzu IR-435 instrument with KBr plates. ^1^H- and ^13^C-NMR spectra were obtained on Bruker DPX 300 spectrometer (Bruker Biospin Co., Stuttgart, Germany) with CDCl_3_ as the solvent and TMS as the internal standard. HR-MS were obtained on a Brüker Apex II mass spectrometer using nitrobenzoyl alcohol and sodium chloride as matrix (ThermoFisher scientific Inc., Waltham, MA, USA).

### 3.2. Synthesis of Spirobisnaphthalene Palmarumycin CP_17_ and Their Methoxy Analogues

#### *5*,*8-Dimethoxytetralone* (**1**)

It was synthesized in 43% overall yield using *p*-dimethoxybenzene as starting material according to the procedures in reference [27].

#### *5*,*8-Dimethoxytetralone methyl enol ether* (**2**)

It was obtained in 93% yield by the reaction of 5,8-dimethoxytetralone and CH(OCH_3_)_3_ in the presence of PPTs according to the protocol in reference [28], which was directly utilized to the next reaction without further purification.

#### *5*,*8*-*Dimethoxy-1*,*2*,*3*,*4-tetrahydrospiro[naphtha-ene-1*,*2′-naphtho-[1,8-de][1,3]dioxine* (**3**)

First 7.0 g (32 mmol) of 5,8-dimethoxytetralone methyl enol ether (**2**), 5.6 g (35 mmol) 1,8-dihydroxynaphthalene, 700 mg (4 mmol) *p*-TsOH and 350 mL toluene were added into a three-necked flask, stirred and heated to reflux under N_2_ atmosphere and kept for 42 h. After the reaction finished, the solution was diluted with 150 mL diethyl ether, washed with NaHCO_3_ solution, dried over anhydrous Na_2_SO_4_, filtered the solid. The organic phase was concentrated under the reduced pressure, the residue was put into a neutral Al_2_O_3_ chromatograph column and eluted with petroleum–ethyl acetate (8:1) to afford a white solid 7.0 g, 64% yield, m.p. 163–164 °C. IR υ (cm^−1^): 3054, 3004, 2946, 2827, 1601, 1580, 1484, 1466, 1411, 1378, 1305. ^1^H-NMR (300 MHz, CDCl_3_) δ: 1.79–1.87 (m, 2H), 2.13–2.17 (m, 2H), 2.77 (t, *J =* 6.5 Hz, 2H), 3.71 (s, 3H), 3.82 (s, 3H), 6.87–6.91 (m, 4H), 7.38–7.47 (m, 4H); ^13^C-NMR (75 MHz, CDCl_3_) δ: 18.93, 24.12, 32.86, 55.87, 57.28, 101.03, 108.77, 111.43, 111.99, 113.02, 119.62, 124.75, 127.28, 129.78, 134.17, 148.04, 150.87, 153.68; ESI-MS(+) *m/z*: 349 [M + H]^+^.

#### *5*,*8-Dimethoxy-2*,*3-dihydrospiro[naphthalene-1*,*2′-naphtho[1*,*8-de][1*,*3]dioxine-4-one* (**5**)

First 1.50 g (4.3 mmol) **3**, 4.5 g PDC, 8.6 g celite and 40 mL benzene were added into a 200 mL flask in an ice-water bath, stirred and injected 6 mL 5–6 M *t*-BuOOH with syringe in 15 m. Then moved the ice-water bath, stirred at room temperature 24 h, and again put 2 mL 5–6 M *t*-BuOOH with syrup, continued to react 24 h in stirring condition. The solution was diluted with ethyl acetate and filtered to remove the solid, the organic phase was washed two times with 25 mL 1 M HCl solution and water, dried over anhydrous Na_2_SO_4_. The organic phase was concentrated under the reduced pressure, the residue was put into a neutral Al_2_O_3_ chromatograph column and eluted with petroleum–ethyl acetate (10:1) to afford a white solid 0.59 g, 65% yield, m.p. 174–176 °C. IR υ (cm^−1^): 3060, 2964, 2936, 2837, 1695, 1606, 1578, 1479, 1410, 1378, 1279, 1154 , 1037; ^1^H-NMR (300 MHz, CDCl_3_) δ: 2.48–2.53 (m, 2H), 2.72–2.77 (m, 2H), 3.75 (s, 3H), 3.91 (s, 3H), 6.94 (d, *J* = 7.2 Hz*,* 2H), 7.12 (d, *J* = 9.2 Hz*,* 1H), 7.28 (d, *J* = 9.2 Hz, 1H), 7.40–7.50 (m, 4H); ^13^C-NMR (75 MHz, CDCl_3_) δ: 31.37, 36.90, 56.91, 57.88, 99.98, 109.02, 112.74, 115.57, 120.26, 121.59, 122.76, 127.41, 129.50, 134.25, 147.38, 152.72, 153.43, 196.07; ESI-MS (+) *m/z*: 385 [M + Na]^+^.

#### *5-Hydroxy-8-methoxy-2*,*3-dihydrospiro[naphthalene-1*,*2′-naphtho-[1*,*8-de][1*,*3]dioxine-4-one* (**6b**)

First 60 mg (0.16 mmol) **5**, 15 mL CHCl_3_, and 0.3 mL (CH_3_)_3_SiI were added into a 25 mL flask, stirred at room temperature 12 h. Then removed the solvent under the reduced pressure, the residue was put into a silica gel chromatograph column and eluted with petroleum–ethyl acetate (4:1) to afford a little yellow solid 42 mg, 73% yield, m.p. 206–207 °C. IR υ (cm^−1^): 3427, 3062, 2959, 2920, 2851, 1693, 1611, 1588, 1474, 1409, 1378, 1295, 1266, 1057; ^1^H-NMR (300 MHz, CDCl_3_) δ: 2.49–2.53 (m, 2H), 2.67–2.71 (m, 2H), 3.92 (s, 3H), 7.06 (dd, *J* = 0.7, 7.5 Hz, 2H), 7.13 (d, *J =* 9.2 Hz, 1H), 7.24 (d, *J* = 9.2 Hz, 1H), 7.47 (t, *J =* 8.4 Hz*,* 2H), 7.59 (dd, *J* = 0.7, 8.4 Hz, 2H), 7.64 (s, 1H); ^13^C-NMR (75 MHz, CDCl_3_) δ: 29.46, 35.60, 56.98, 102.45, 110.50, 113.59, 116.93, 121.50, 121.77, 123.67, 124.81, 127.57, 134.18, 146.34, 148.92, 153.71, 194.94. HR-ESI-MS: calcd for [M + H]^+^ C_21_H_17_O_5_: 349.1070, found: 349.1071.

#### *8-Hydroxynaphthalen-1-yl-4*,*5*,*8-trihydroxynaphthalene ether* (**7**)

First 0.72 g (2 mmol) **5**, First 60 mL CHCl_3_, and 3.0 mL (CH_3_)_3_SiI were added into a 150 mL flask, stirred at 50 °C in an oil bath for 24 h. Then added 50 mL CHCl_3_, and washed with 5% Na_2_S_2_O_3_ solution and water, removed the solvent under the reduced pressure, the residue was put into a silica gel chromatograph column and eluted with petroleum–ethyl acetate (10:1) to afford a little yellow solid 0.31 g, 44% yield, m.p. 206–207 °C. ^1^H-NMR (300 MHz, CDCl_3_) δ: 6.98 (dd, *J =* 1.1, 7.6 Hz, 1H), 7.25–7.44 (m, 6H), 7.7.51–7.58 (m, 2H), 7.77 (d, *J =* 8.8 Hz*,* 1H), 8.88 (s, 1H), 12.42 (s, 1H). ESI-MS (+) *m/z*: 335 [M + H]^+^.

#### *8-((8-Hydroxynaphthalen-1-yl)oxy)naphthalene-1,4,5-triol* (**8**)

In a 25 mL flask, 250 mg **5** (0.69 mmol) was dissolved in 10 mL methanol, 27 mg NaBH_4_ (0.7 mmol) was added into the flask at ambient temperature and stirred for 30 min. The solvent was removed *in vacuo*, 20 mL water was added, and extracted with ethyl acetate (3 × 20 mL). The organic phase was combined and dried over anhydrous Na_2_SO_4_, then the solvent was removed under reduced pressure to produce a white solid 242 mg, yield 97%, m.p. 189–190 °C. IR υ (cm^−1^): 3053, 3004, 2934, 2827, 1603, 1582, 1486, 1411, 1380, 1307, 1273, 1261, 1061, 906; ^1^H-NMR (300 MHz, CDCl_3_) δ: 2.02 (d, *J* = 15.3 Hz, 1H), 2.15–2.11 (m, 2H), 2.34 (dd, *J* = 3.0, 12.0 Hz, 1H), 3.06 (br, 1H), 3.71 (s, 3H), 3.89 (s, 3H), 5.11 (t, *J* = 4.5 Hz, 1H), 6.87–6.93 (m, 2H), 6.98 (d, *J* = 1.2 Hz, 2H), 7.37–7.48 (m, 4H); ^13^C-NMR (75 MHz, CDCl_3_): δ: 26.10, 27.40, 55.00, 57.50, 63.10, 100.61, 108.80, 112.64, 114.59, 119.78, 124.70, 127.29, 147.98, 151.40, 153.70. HRMS: calcd for C_22_H_21_O_5_, 365.1384, found: [M + H]^+^, 365.1382.

#### *5*,*8-Dihydroxy-1*,*2*,*3*,*4-tetrahydrospiro[naphthalene-1*,*2′-naphtho-[1*,*8-de][1*,*3]dioxine* (**9a**) and *5-Hydroxy-8-methoxy-1*,*2*,*3*,*4-tetrahydrospiro[naphthalene-1*,*2′-naphtho-[1*,*8-de][1*,*3]dioxine* (**9b**)

First 1.0 g (2.9 mmol) **3**, 125 mL CHCl_3_, and 4.3 mL (CH_3_)_3_SiI (30 mmol) were added into a 250 mL flask, stirred at 50 °C in an oil bath for 72 h. Then added 10 mL CH_3_OH and stirred 0.5 h at room temperature, diluted with 50 mL CHCl_3_, washed with 5% Na_2_S_2_O_3_ solution and water, dried over anhydrous Na_2_SO_4_. The solvent was removed under the reduced pressure, the residue was put into a silica gel chromatograph column and eluted with petroleum–ethyl acetate (5:1) to afford a white solid **9a** 0.79 g, 86% yield, m.p. 170–171 °C. IR υ (cm^−1^): 3057, 2928, 1610, 1579, 1466, 1409, 1388, 1346. ^1^H-NMR (300 MHz, CDCl_3_) δ: 1.87–1.94 (m, 2H), 2.15–2.19 (m, 2H), 2.77 (t, *J* = 6.4 Hz, 2H), 4.44 (s, 1H), 6.80 (dd, *J* = 8.7, 11.0 Hz, 2H), 6.99 (d, *J =* 0.9 Hz, 2H), 7.02 (s, 1H), 7.45 (dd, *J =* 7.5, 8.4 Hz, 2H), 7.55 (dd, *J =* 0.9, 8.4 Hz, 2H); ^13^C-NMR (75 MHz, CDCl_3_) δ:18.72, 23.55, 30.79, 103.29, 110.39, 113.91, 115.70, 117.56, 120.33, 121.22, 125.78, 127.47, 134.14, 146.02, 147.05, 150.38. When the quantity of (CH_3_)_3_SiI was 5 eq. molar of **3** without changing the other condition, a little yellow solid **9b** was obtained in 58% yield, 164–166 °C. IR υ (cm^−1^): 3440, 3060, 2998, 2983, 2944, 2927, 2864, 2831, 1606, 1582, 1477, 1466, 1410, 1378, 1263; ^1^H-NMR (300 MHz, CDCl_3_) δ: 1.83–1.89 (m, 2H), 2.13–2.17 (m, 2H), 2.78 (t, *J* = 6.1 Hz, 2H), 3.82 (s, 3H), 6.88 (dd, *J* = 8.9, 10.8 Hz, 2H), 6.99–7.02 (m, 3H), 7.45 (dd, *J* = 7.6, 8.4 Hz, 2H), 7.55 (dd, *J* = 0.8, 8.4 Hz, 2H); HR-ESI-MS: calcd for [M + H]^+^ C_21_H_19_O_4_: 335.1278, found: 335.1278.

#### *5*,*8-Diacetoxy-1*,*2*,*3*,*4-tetrahydrospiro[naphthalene-1*,*2’-naphtho-[1*,*8-de][1*,*3]dioxine* (**10a**)

First 1.95 g (6.1 mmol) **9a**, 1.8 mL (12.9 mmol) Et_3_N and 50 mL CH_2_Cl_2_ were added into a 150 mL flask and sealed, injected 1.8 mL Ac_2_O with syringe in 1 h, and stirred another 1 h at room temperature. Then removed the solvent, the residue was put into a silica gel chromatograph column and eluted with petroleum–ethyl acetate (6:1) to afford a white solid **10a** 1.65 g, 68% yield, m.p. 151–153 °C. IR υ (cm^−1^): 2943, 2878, 1763, 1693, 1640, 1469, 1375, 1195; ^1^H-NMR (300 MHz, CDCl_3_) δ: 1.75 (s, 3H), 1.84–1.88 (m, 2H), 2.14–2.18 (m, 2H), 2.34 (s, 3H), 2.69 (t, *J* = 12.2 Hz, 2H), 6.92 (dd, *J =* 1.0 Hz, 7.2 Hz, 2H), 7.04 (d, *J* = 8.7 Hz, 1H), 7.17 (d, *J =* 8.7 Hz, 1H), 7.40–7.51 (m, 4H); ^13^C-NMR (75MHz, CDCl_3_) δ: 18.62, 20.59, 20.74, 24.15, 31.92, 100.10, 109.14, 112.68, 120.28, 123.25, 123.56, 127.44, 128.17, 133.09, 134.17, 145.98, 147.28, 147.70, 168.74, 169.61; HR-ESI-MS: calcd for [M + H]^+^ C_24_H_21_O_6_: 405.1333, found: 405.1332.

#### *5-Acetoxy-8-methoxy-1*,*2*,*3*,*4-tetrahydrospiro[naphthalene-1*,*2′-naphtho-[1*,*8-de][1*,*3]dioxine* (**10b**)

When 140 mg **9b** was used as raw material to react in the similar way as **9a** without changing the other condition, a white solid **10b** 150 mg was obtained in 95% yield, m.p. 148–150 °C. IR υ (cm^−1^): 3055, 2952, 2936, 2837, 1760, 1603, 1583, 1477, 1438, 1413, 1380, 1276, 1209, 1065, 1021; ^1^H-NMR (300 MHz, CDCl_3_) δ: 1.56 (s, 3H), 1.81–1.89 (m, 2H), 2.12–2.16 (m, 2H), 2.77 (t, *J* = 6.3 Hz, 2H), 3.86 (s, 3H), 6.90–6.93 (m, 3H), 6.99 (d, *J =* 5.8 Hz, 1H), 7.39–7.49 (m, 4H); ^13^C-NMR (75 MHz, CDCl_3_) δ: 18.77, 20.62, 23.75, 31.97, 55.78, 100.49, 109.08, 110.83, 112.77, 120.14, 122.58, 127.41, 129.67, 134.18, 142.89, 147.48, 154.53, 170.23. HR-ESI-MS: calcd for [M + H]^+^ C_23_H_21_O_5_: 377.1383, found: 377.1384. 

#### *5*,*8-Diacetoxy-2*,*3-dihydrospiro[naphthalene-1*,*2′-naphtho[1*,*8-de][1*,*3]dioxine-4-one* (**11a**) *and 5-Acetoxy-8-methoxy-2*,*3-dihydrospiro[naphthalene-1*,*2′-naphtho[1*,*8-de][1*,*3]dioxine-4-one* (**11b**)

First 210 mg (0.56 mmol) **10b**, 0.4 g PDC, 1.2 g celite and 6 mL benzene were added into a 50 mL flask in a ice-water bath, stirred and injected 1 mL 5–6 M *t*-BuOOH with syringe in 15 min. Then moved the ice-water bath, stirred at room temperature 24 h, and again injected 0.5 mL 5–6 M *t*-BuOOH with syringe, continued to react in stirring condition 24 h. The solution was diluted with ethyl acetate and filtered to remove the solids, the organic phase was washed two times with 10 mL 1 M HCl solution and water, dried over anhydrous Na_2_SO_4_. The organic phase was concentrated under the reduced pressure, the residue was put into a neutral Al_2_O_3_ chromatograph column and eluted with petroleum–ethyl acetate (5:1) to afford a white solid **11b** 152 mg, 69% yield, m.p. 148–150 °C. IR υ (cm^−1^): 2925, 2853, 1748, 1583, 1478, 1412, 1379, 1310, 1276, 1113; ^1^H-NMR (300 MHz, CDCl_3_) δ: 1.83 (s, 3H), 2.48–2.52 (m, 2H), 2.72–2.77 (m, 2H), 3.97 (s, 3H), 6.96 (dd, *J* = 1.0, 7.4 Hz, 2H), 7.18 (d, *J =* 9.3 Hz, 1H), 7.35 (d, *J* = 9.1 Hz, 1H), 7.45 (dd, *J* = 7.4, 8.3 Hz, 2H), 7.53 (dd, *J = 1*.0, 8.5 Hz, 2H); ^13^C-NMR (75 MHz, CDCl_3_) δ: 20.51, 30.50, 36.40, 56.71, 99.41, 109.33, 112.49, 114.70, 120.78, 122.24, 127.54, 131.30, 132.59, 134.19, 141.78, 146.76, 157.28, 169.92,194.86. HR-ESI-MS: calcd for [M + H]^+^ C_23_H_19_O_6_: 391.1176, found: 391.1177. In the similar way, **11a** was obtained in 55% yield, m.p. 136–137 °C. IR υ (KBr): 2945, 2859, 1750, 1585, 1482, 1408, 1375, 1304, 1278, 1115 cm^−1^; ^1^H-NMR ((300 MHz, CDCl_3_) δ: 1.81 (s, 3H), 2.28 (s, 3H), 2.49–2.53 (m, 2H), 2.71–2.77 (m, 2H), 6.95 (dd, *J* = 1.0 Hz, 7.4 Hz, 2H), 7.18 (d, *J =* 9.3 Hz, 1H), 7.36 (d, *J* = 9.1 Hz, 1H), 7.47 (dd, *J* = 7.4 Hz, 8.3 Hz, 2H), 7.55 (dd, *J =* 1.0 Hz, 8.5 Hz, 2H); ^13^C-NMR (75 MHz, CDCl_3_) δ: 20.50, 21.81, 30.61, 36.52, 99.52, 109.43, 112.51, 114.68, 120.76, 122.25, 127.54, 131.35, 132.64, 134.22, 141.83, 146.79, 157.26, 169.95, 194.88. HR-MS (ESI): calcd for C_24_H_19_O_7_ [M + H]^+^: 419.1131, found: 419.1132.

#### *5*,*8-Dihydroxy-2*,*3-dihydrospiro[naphthalene-1*,*2′-naphtho[1*,*8-de][1*,*3]dioxine-4-one* (Palmarumycin CP_17_, **6a**) and *5-Hydroxy-8-methoxy-2*,*3-di-hydrospiro[naphthalene-1*,*2′-naphtho[1*,*8-de][1*,*3]-dioxine-4-one* (**6b**)

The 5 mL methanol solution of 168 mg (0.40 mmol) **11a** was added a 25 mL flask, stirred and added 9 mL 1% NaOH solution at room temperature to react 1 h. Then adjusted pH value to 5–6 with 1 M HCl, removed the methanol under the reduced pressure. Extracted with ethyl acetate (3 × 10 mL) and dried over anhydrous Na_2_SO_4_. The solvent was removed from the organic phase to afford a white solid 6a 122 mg, 91% yield, m.p. 173–174 °C. IR υ (cm^−1^): 3435, 3064, 2969, 2924, 2856, 1695, 1610, 1585, 1473, 1407, 1376, 1290, 1264, 1058; ^1^H-NMR (300 MHz, CDCl_3_) δ: 2.49–2.53 (m, 2H), 2.74–2.78 (m, 2H), 7.04–7.07 (m, 3H), 7.24–7.27 (m, 1H), 7.48 (t, *J =* 7.4 Hz*,* 2H), 7.59 (d, *J* = 7.5 Hz, 2H), 7.65 (s, 1H), 12.36 (s, 1H); ^13^C-NMR (75 MHz, CDCl_3_) δ: 28.98, 33.85, 102.06, 110.46, 113.53, 114.49, 119.49, 121.85, 127.61, 128.87, 134.22, 146.29, 147.68, 157.20, 202.41. HR-MS (ESI): calcd for [M − H]^−^ C_21_H_17_O_5_: 333.0768, found: 333.0769. These data were consistent with that of naturally occurring compounds [19]. In the similar way, 145 mg (0.37 mmol) **11b** was transferred into 121 mg of **6b** in 93% yield, the m.p., ^1^H-NMR, ^13^C-NMR and MS data were identical with that of the demethylation product of compound **5**.

#### *7-Methoxytetralone* (**13**) and *6*,*7*-*Dimethoxytetralone* (**14**)

They were prepared by the similar methods [27] as **1** using methoxybenzene and 1,2-dimethoxybenzene as the starting materials. Compound **13**, a white solid, 85% yield, m.p. 59–60 °C. ^1^H NMR (300 MHz, CDCl_3_) δ: 2.07–2.16 (m, 2H), 2.64 (t, *J* = 6.4 Hz, 2H), 2.90 (t, *J* = 6.4 Hz, 2H), 3.83 (s, 3H), 7.05 (dd, *J =* 2.8, 8.7 Hz, 1H), 7.17 (d, *J =* 8.7 Hz, 1H), 7.52 (d, *J =* 2.8 Hz, 1H); ESI-MS *m/z*: 177 [M + H]^+^, 199 [M + Na]^+^. Compound **14**, a white solid, 75% yield, m.p. 94–95 °C. ^1^H-NMR (300 MHz, CDCl_3_) δ: 2.08–2.17 (m, 2H), 2.60 (t, *J* = 6.5 Hz, 2H), 2.90 (t, *J* = 6.3 Hz, 2H), 3.92 (s, 3H), 3.94 (s, 3H), 6.68 (s, 1H), 7.52 (s, 1H); ESI-MS *m/z*: 207 [M + H]^+^, 229 [M + Na]^+^. 6-Methoxytetralone (**12**) was purchased from Acros Organic Co.

#### *6-Methoxy-3,4-dihydro-2H-spironaphthalene-1,2′-naphtho[1*,*8-de][1*,*3]dioxine* (**15**), *7-Methoxy-3,4-dihydro-2H-spironaphthalene-1,2′-naphtho[1*,*8-de][1*,*3]dioxine* (**16**) and *6,7-Dimethoxy-3,4-dihydro-2H-spironaphthaene-1,2′-naphtho[1*,*8-de][1*,*3]dioxine* (**17**)

These enol methyl ether intermediates were prepared in the similar methods as **2** using **12**, **13** and **14** as raw materials, respectively. Then compounds **15**, **16** and **17** were obtained by reaction of these enol methyl ethers with 1,8-dihydroxynaphthalene in the catalysis of *p*-TsOH in toluene as compound **3**. Compound **15**, a white solid, 61% yield, m.p. 146–147 °C. ^1^H-NMR (300 MHz, CDCl_3_) δ: 1.88–1.96 (m, 2H), 2.12–2.16 (m, 2H), 2.88 (t, *J* = 6.2 Hz, 2H), 3.83 (s, 3H), 6.72 (d, *J* = 2.6 Hz, 1H), 6.88–6.92 (m, 3H), 7.39–7.50 (m, 4H), 7.77 (d, *J* = 8.7 Hz, 1H); ^13^C-NMR (75 MHz, CDCl_3_) δ: 19.81, 29.70, 31.11, 55.31, 100.69, 109.25, 112.86, 113.15, 113.69, 120.16, 127.35, 127.87, 129.05, 134.18, 139.77, 148.44, 160.22; ESI-MS: *m/z* 319 [M + H]^+^. Compound **16**, a white solid, 65% yield, m.p. 105–107 °C. ^1^H-NMR (300 MHz, CDCl_3_) δ: 1.89–1.95 (m, 2H), 2.13–2.17 (m, 2H), 2.85 (t, *J* = 6.3 Hz, 2H), 3.81 (s, 3H), 6.92–6.97 (m, 3H), 7.14 (d, *J* = 8.5 Hz, 1H), 7.36 (d, *J* = 2.7 Hz, 1H), 7.40–7.51 (m, 4H); ^13C-^NMR (75 MHz, CDCl_3_) δ: 28.56, 29.69, 31.98, 55.42, 100.68, 109.35, 110.84, 113.70, 117.26, 120.27, 127.38, 129.81, 130.20, 134.20, 135.92, 148.23, 158.37; ESI-MS: *m/z* 319 [M + H]^+^. Compound **17**, a white solid, 67% yield, m.p. 143–145 °C. ^1^H-NMR (CDCl_3_) δ: 1.90–1.96 (m , 2H), 2.12–2.16 (m, 2H), 2.84 (t, *J* = 6.0 Hz, 2H), 3.89 (s, 3H), 3.91 (s, 3H), 6.67 (s, 1H), 6.92–6.95 (m, 1H), 7.29 (d, *J =* 6.0 Hz, 1H), 7.41–7.51 (m, 4H); ^13^C-NMR (75MHz, CDCl_3_) δ: 19.96, 29.06, 31.01, 55.94, 55.95, 100.82, 109.34, 109.52, 110.71, 113.90, 120.23, 127.13, 127.28, 130.99, 134.20, 146.02, 148.38, 150.08; ESI-MS: *m/z* 349 [M + H]^+^.

#### *6-Methoxy-2*,*3-dihydrospiro[naphthalene-1*,*2′-naphtho[1*,*8-de][1*,*3]dioxine-4-one* (**18**), *7-Methoxy-2*,*3-dihydrospiro[naphthalene-1*,*2′-naphtho-[1*,*8-de][1*,*3]dioxine-4-one* (**19**) and *6*,*7-Dimethoxy-2*,*3-di-hydrospiro[naphthalene-1*,*2′-naphtho-[1*,*8-de][1*,*3]dioxine-4-one* (**20**)

The benzylic oxidation of three intermediates **15**–**17** by PDC and *t*-BuOOH as **5** afforded **18**, **19** and **20**. Compound **18**, a white solid, 68% yield, m.p. 145–146 °C. ^1^H-NMR (300 MHz, CDCl_3_) δ: 2.51 (t, *J =* 6.3 Hz, 2H), 2.80 (t, *J =* 6.3 Hz, 2H), 3.91 (s, 3H), 6.96 (dd, *J =* 7.4, 1.0 Hz, 2H), 7.24–7.28 (m, 1H), 7.42–7.59 (m, 5H), 7.89 (d, *J* = 8.7 Hz, 1H); ^13^C-NMR (75 MHz, CDCl_3_) δ: 29.85, 34.42, 55.66, 98.80, 109.31, 109.35, 113.42, 120.72, 121.93, 127.48, 127.93, 132.95, 134.20, 147.73, 160.87, 196.19; ESI-MS *m/z*: 333 [M + H]^+^. Compound **19**, a white solid, 63% yield, m.p. 147–148 °C. ^1^H-NMR (300 MHz, CDCl_3_) δ: 2.50 (t, *J =* 6.3 Hz, 2H), 2.75 (t, *J =* 6.3 Hz, 2H), 3.90 (s, 3H), 6.98 (d, *J =* 7.4 Hz, 2H), 7.08 (dd, *J =* 2.6, 8.7 Hz, 1H), 7.43–7.55 (m, 5H), 8.10 (d, *J* = 8.7 Hz, 1H); ^13^C-NMR (75 MHz, CDCl_3_) δ: 29.82, 34.95, 55.69, 98.70, 109.45, 109.91, 113.42, 116.77, 120.83, 125.13, 129.57, 134.20, 142.69, 147.52, 164.43, 194.99; ESI-MS *m/z*: 333 [M + H]^+^. Compound **20**, a white solid, 49% yield, m.p. 148–149 °C. ^1^H-NMR (300 MHz, CDCl_3_) δ: 2.51 (t, *J =* 6.3 Hz, 2H), 2.77 (t, *J =* 6.3 Hz, 2H), 3.99 (s, 3H), 4.01 (s, 3H), 6.99 (dd, *J =* 7.4, 1.0 Hz, 2H), 7.41–7.59 (m, 6H); ^13^C-NMR (75 MHz, CDCl_3_) δ: 30.03, 34.12, 56.23, 56.32, 98.88, 107.84, 108.29, 109.49, 113.46, 120.83, 125.48, 127.52, 134.25, 135.06, 147.70, 150.43, 154.21, 195.15; ESI-MS *m/z*: 363 [M + H]^+^.

#### *8-Hydroxynaphthalen-1-yl-4-hydroxy-6-methoxynaphthalene ether* (**21**), *8-Hydroxynaphthalen-1-yl-4-hydroxy-7-methoxynaphthalene ether* (**22**) and *8-Hydroxy-naphthalen-1-yl-4,7-dimethoxynaphthalene ether* (**23**)

The (CH_3_)_3_SiI demethylation of compound **18** was carried out as **5** at room temperature to afford compound **21** as a little yellow oily liquid, yield 95%. ^1^H-NMR (300 MHz, CDCl_3_) δ: 3.97 (s, 3H), 5.50 (brs, 1H), 6.43 (dd, *J* = 7.8, 1.0 Hz, 1H), 6.81 (d, *J* = 8.1Hz, 1H), 7.00–7.15 (m, 4H), 7.36–7.53 (m, 4H), 7.85 (d, *J* = 8.7 Hz, 1H), 9.32 (brs, 1H); ^13^C-NMR (75 MHz, CDCl_3_) δ: 55.44, 100.76, 108.54, 108.69, 110.74, 115.11, 115.44, 119.22, 120.20, 122.79, 123.42, 123.50, 125.62, 126.82, 127.79, 136.98, 143.46, 148.53, 154.12, 156.52, 158.12. ESI-MS *m/z*: 355 [M + Na]^+^. Similarly, compounds **22** and **23** were obtained using **19** as starting material in same process. Compound **22**, a little yellow solid, yield 48%, m.p. 128–130 °C. ^1^H-NMR (300 MHz, CDCl_3_) δ: 3.73 (s, 3H), 5.76 (brs, 1H), 6.49 (dd, *J* = 7.8, 1.0 Hz, 1H), 6.66 (d, *J* = 8.1 Hz, 1H), 7.02 (dd, *J* = 7.4, 1.8 Hz, 1H), 7.10–7.25 (m, 4H), 7.37–7.48 (m, 3H), 8.16 (d, *J* = 8.7 Hz, 1H), 9.39 (brs, 1H); ^13^C-NMR (75 MHz, CDCl_3_) δ: 55.35, 99.81, 105.96, 108.83, 110.70, 115.24, 118.77, 119.27, 120.71, 122.85, 124.28, 125.72, 127.76, 129.61, 136.96, 142.59, 149.83, 154.06, 156.28, 158.98. ESI-MS *m/z*: 355 [M + Na]^+^. Compound **23**, a little yellow solid, m.p. 138–139 °C, yield 35%. ^1^H-NMR (300 MHz, CDCl_3_) δ: 3.73 (s, 3H), 4.01 (s, 3H), 6.49 (dd, *J* = 7.8, 1.0 Hz, 1H), 6.67 (d, *J* = 8.1 Hz, 1H), 7.02 (dd, *J* = 7.4, 1.8 Hz, 1H), 7.10–7.25 (m, 4H), 7.37–7.48 (m, 3H), 8.23 (d, *J* = 8.7 Hz, 1H), 9.37 (brs, 1H). ESI-MS *m/z*: 369 [M + Na]^+^. 

^1^H-, ^13^C-NMR and HR-MS spectra of compounds **1**–**23** can be found in the Supplementary Materials.

### 3.3. Bioassay of Spirobisnaphthalene Palmarumycin CP_17_ and Its Methoxy Analogues

Antifungal activities of the compounds against *P. piricola*, *B. cinerea*, *A.solani*, *C. arachidicolahori*, *F. oxysporurm*, *F. graminearum* and *P. asaragisace* were evaluated using the mycelial growth rate test [38]. The culture media with known concentration of the test compounds were obtained by mixing the solution in acetone with potato dextrose agar (PDA), on which fungus cakes were placed. The blank test was made using acetone and carbendazim was used as the positive control. The culture was incubated at 25 ± 0.5 °C. Three replicates were performed. After the mycelia in the blank grew completely, the diameter of the mycelia was measured, and the inhibition rate was calculated according to the formula in reference [38].

## 4. Conclusions

The total syntheses of palmarumycin CP_17_ **6a** and its 8-methoxy analogue **6b** were carried out in nine steps, using *p*-dimethoxybenzene as the starting material, with 4.9% and 5.8% overall yields, respectively. The direct acetalization of enol ether and 1,8-dihydroxy naphthalene, and benzylic oxidation were used as the key steps in constructing the core spiroketal and the carbonyl group at C_4_. The spirobisnaphthalenes **18**–**20** were also prepared with a similar protocol, but their demethylation with (CH_3_)_3_SiI at ambient temperature resulted in ring A aromatization and acetal cleavage to afford binaphthyl ethers. The EC_50_ values of **5** and **9b** against *P. piricola* and **11b** against *R. solani* were 9.34, 12.35 and 11.18 µg/mL, which showed their strong antifungal activities on these phytopathogens.

## Figures and Tables

**Table 1:** The antifungal activities of compounds against phytopathogens (inhibitory rate, %) at 50 µg/mL ^a^.

Compd.	*R.S*	*P.P*	*B.C*	*F.O*	*P.A*	*F.G*	*C.A*
**3**	44.5	33.6	50.5	10.3	21.7	18.6	49.7
**5**	54.5	73.7	61.2	13.0	33.3	18.6	49.7
**8**	48.9	43.5	60.5	16.0	38.3	23.0	51.4
**9a**	52.1	57.0	68.4	16.3	23.0	13.9	44.7
**9b**	51.1	75.0	57.9	14.0	29.8	11.6	54.8
**10a**	55.3	63.5	69.0	21.7	36.7	18.6	58.0
**10b**	42.3	50.6	67.2	14.9	26.5	18.6	53.1
**11b**	71.3	0	67.1	10.0	2.7	23.0	59.0
**6a**	48.9	43.5	60.5	16.0	38.3	23.0	51.4
**6b**	52.4	64.7	60.5	21.3	39.0	41.9	50.6

^a^ *R.S*: *Rhizoctonia solani*; *P.P*: *Physalospora piricola*; *B.C*: *Botrytis cinerea*; *F.O*: *Fusarium oxysporum*; *P.A*: *Phomopsis asaragisace*; *F.G*: *Fusarium graminearum*; *C.A*: *Cercospora arachidicolahori*. The data are the mean measurements that were calculated from the three replicates with 0% ± 5% errors.

**Table 2:** The EC_50_ values for compounds **5**, **9b** and **11b** against different phytopathogens.

Compd.	Fungi	Regression Eq.	EC_50_ (µg/mL)	Correlation efficient (*R*^2^)
**5**	*P. piricola*	Y = −1.278 + 1.282X	9.34	0.9604
**9b**	*P. piricola*	Y = −3.014 + 2.760X	12.35	0.9525
**11b**	*R. solani*	Y = −2.076 + 1.980X	11.18	0.9645

## Supplementary Materials

Supplementary materials can be accessed at: http://www.mdpi.com/1420-3049/21/5/600/s1.
