# Supplementary material for: Total Synthesis and Antifungal Activity of Palmarumycin CP17 and Its Methoxy Analogues
Source: Molecules. 2016 May 7;21(5):600. doi: 10.3390/molecules21050600 (PMC6274023; doi:10.3390/molecules21050600)
Supplement: Supplementary file 1 [file molecules-21-00600-s001.pdf]

Supplementary Materials: Total Synthesis and Antifungal Activity of Palmarumycin CP17 and Its Methoxy Analogues

Ruina Wang, Guoyue Liu, Mingyan Yang, Mingan Wang and Ligang Zhou

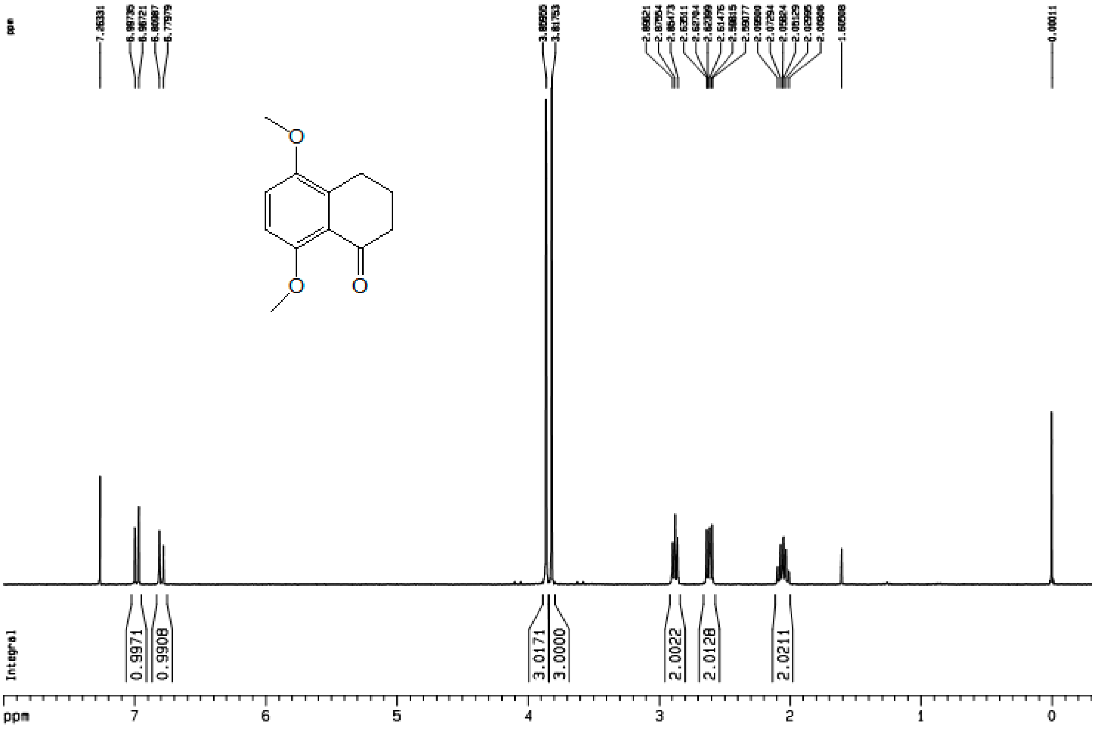

Figure S1. ^{1}H NMR of compound **1**.

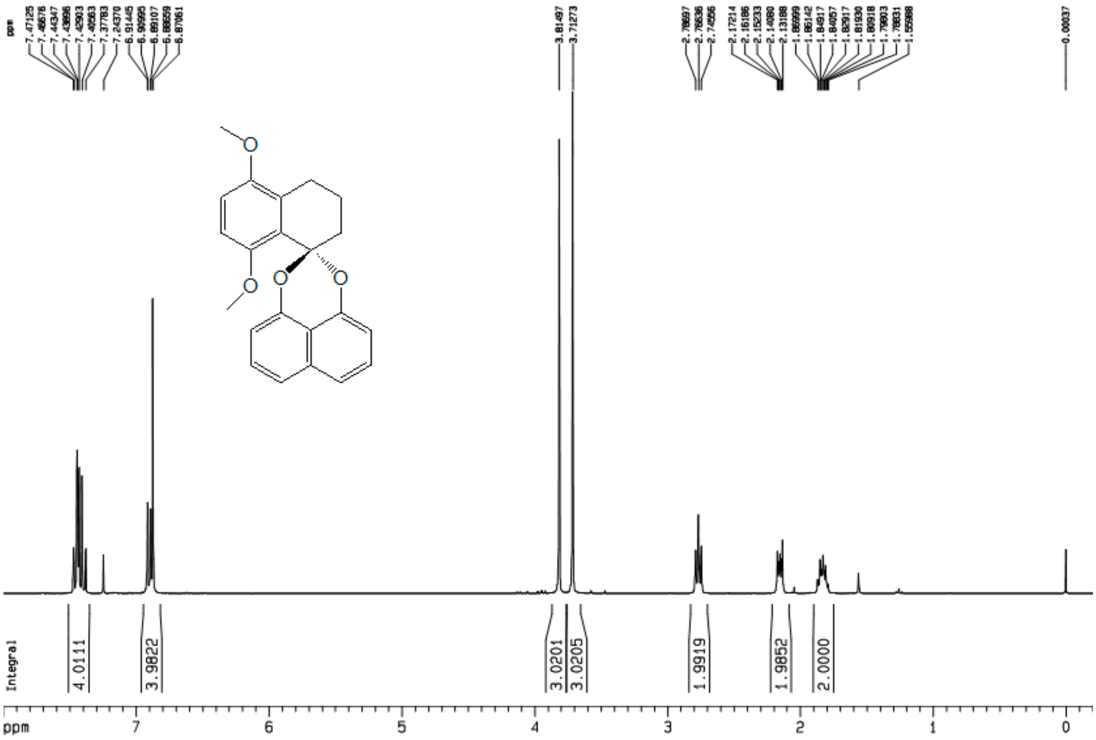

Figure S2. ^{1}H NMR of compound **3**.

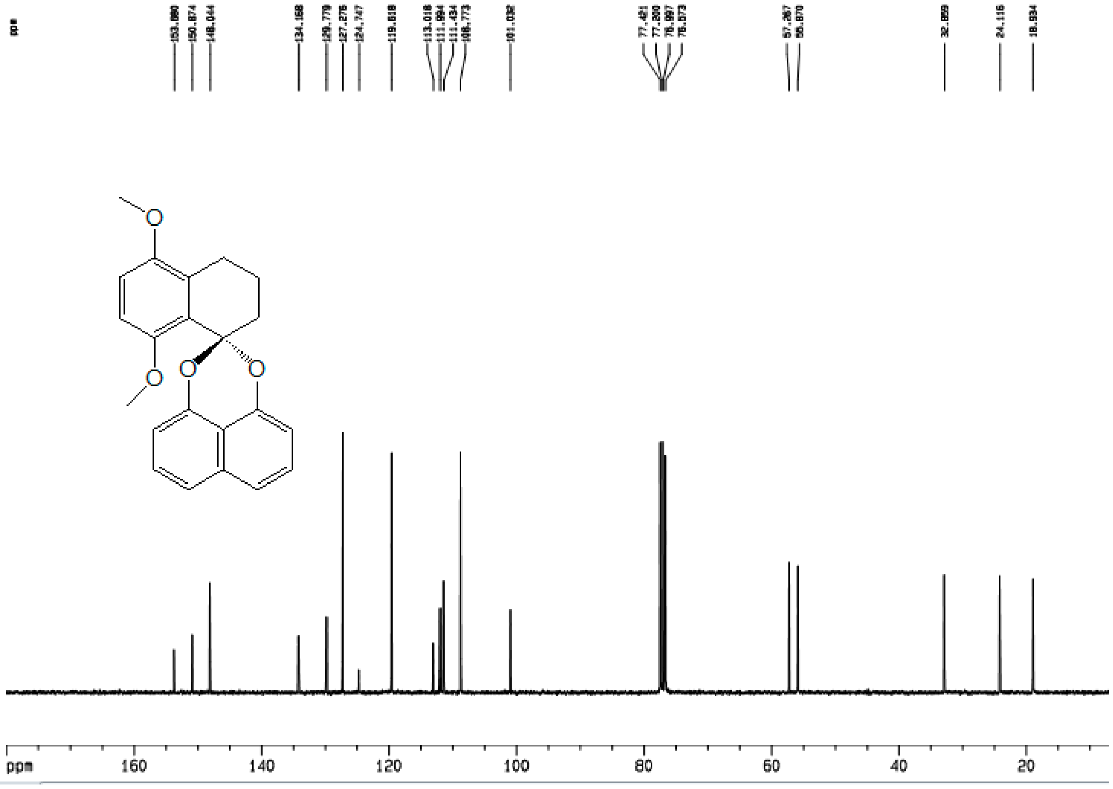

Figure S3. ^{13}C NMR of compound **3**.

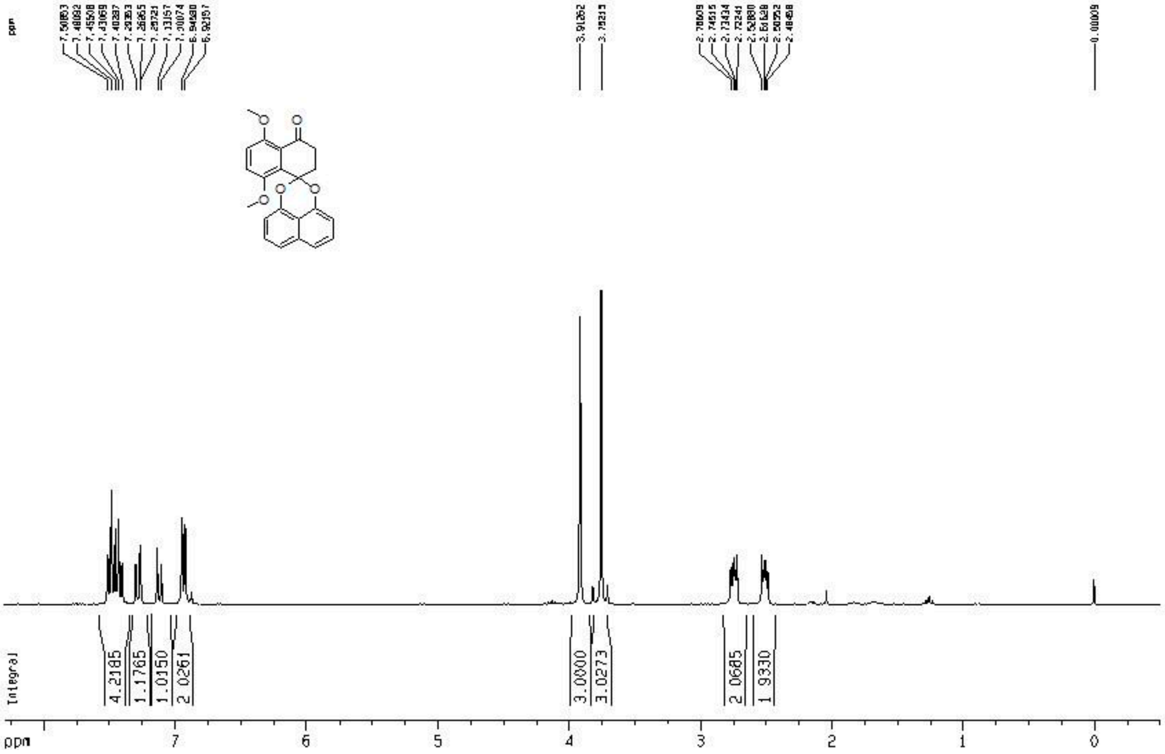

Figure S4. ^{1}H NMR of compound **5**.

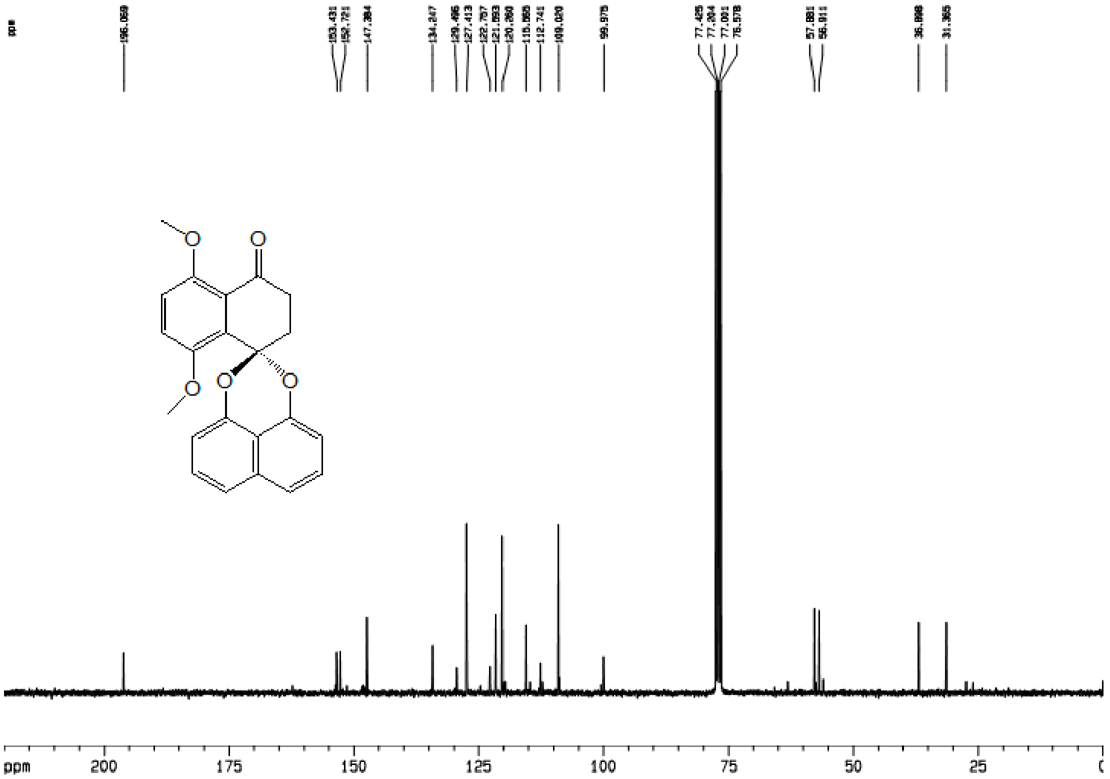

Figure S5. ^{13}C NMR of compound **5**.

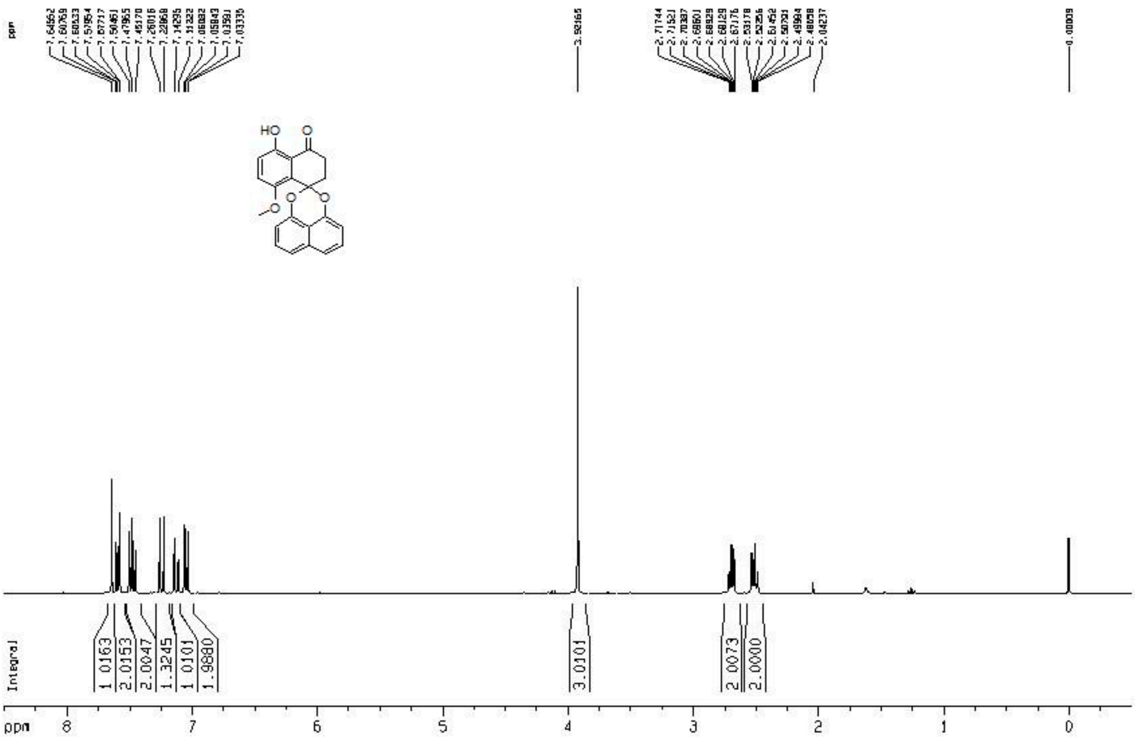

Figure S6. ^{1}H NMR of compound **6b**

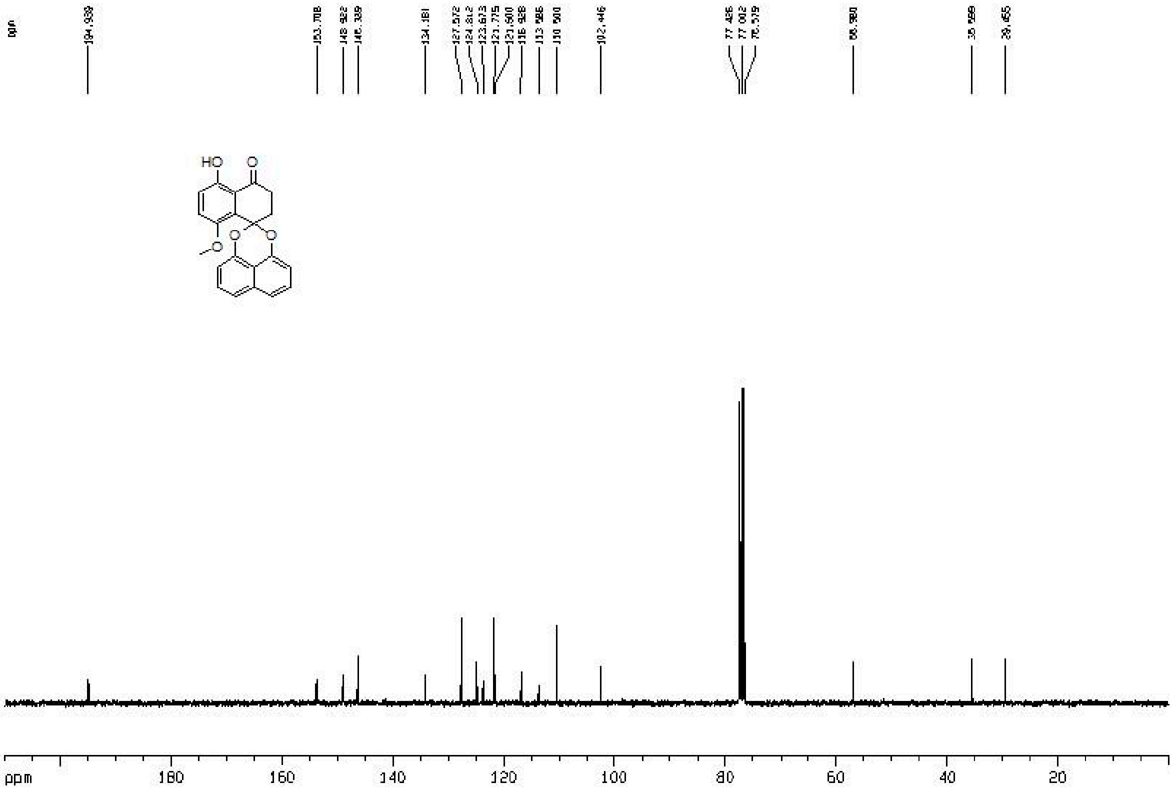

Figure S7. ^{13}C NMR of compound **6b**

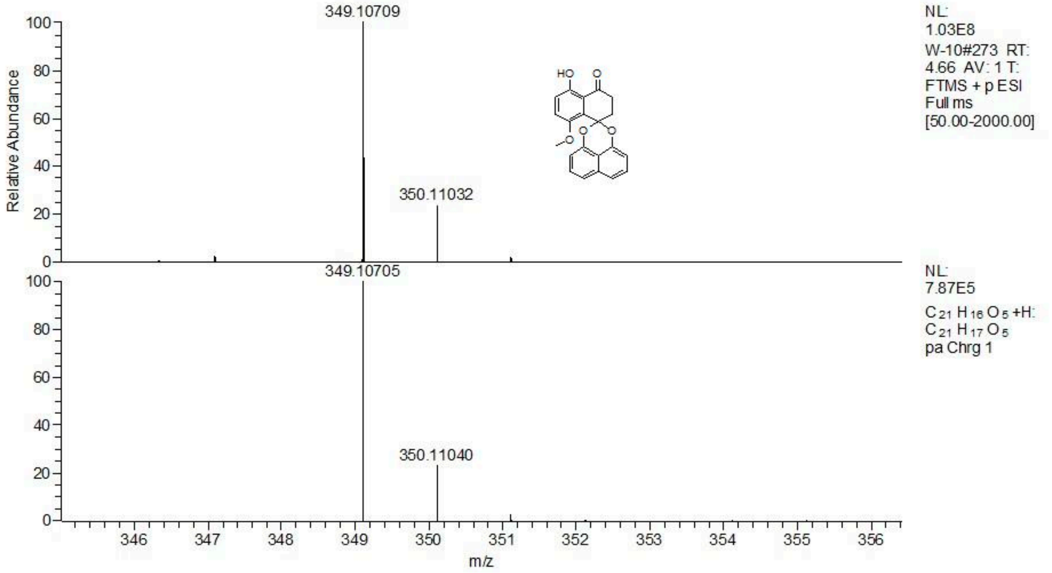

Figure S8. HR-MS (ESI) of compound **6b**.

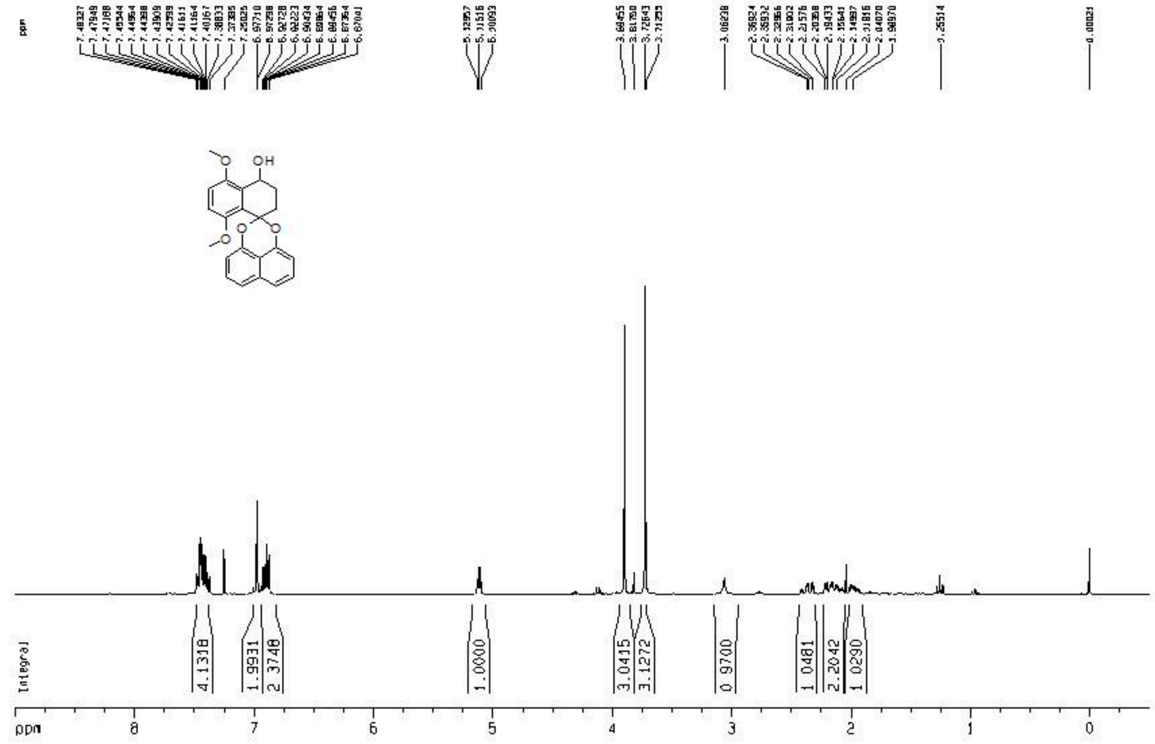

Figure S9. ^{1}H NMR of compound **8**.

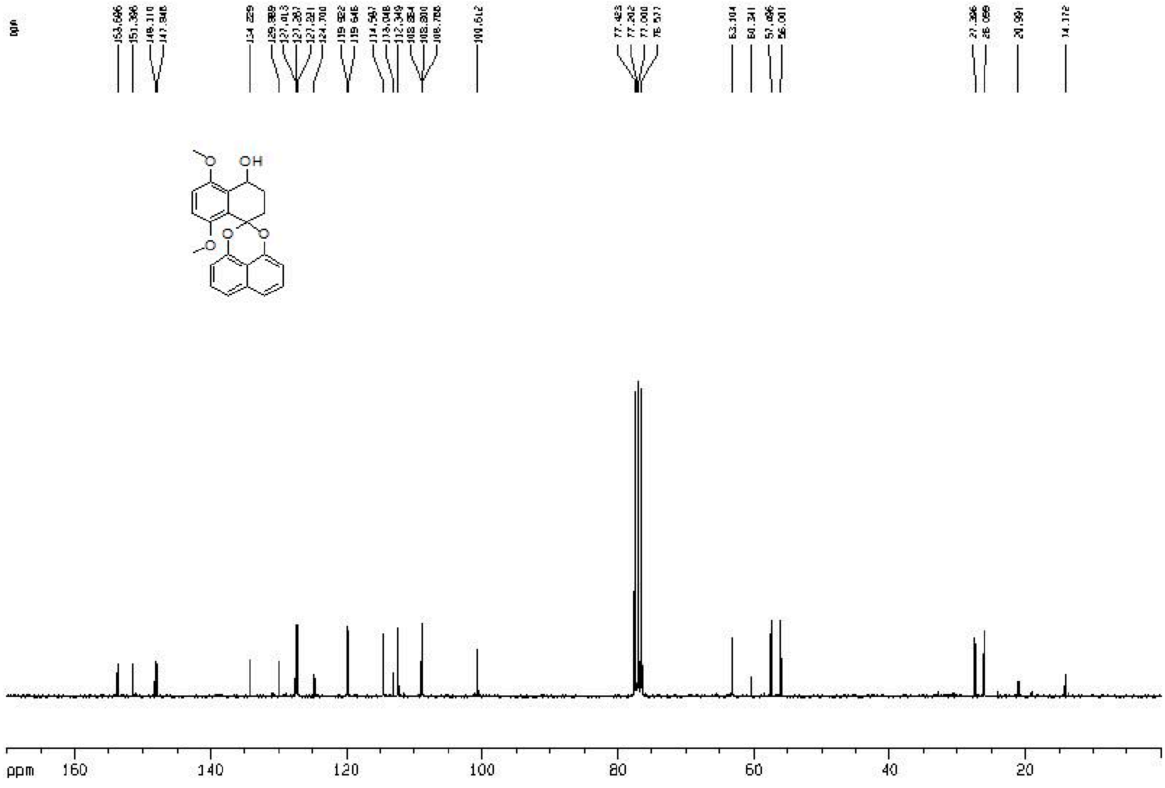

Figure S10. ^{13}C NMR of compound **8**.

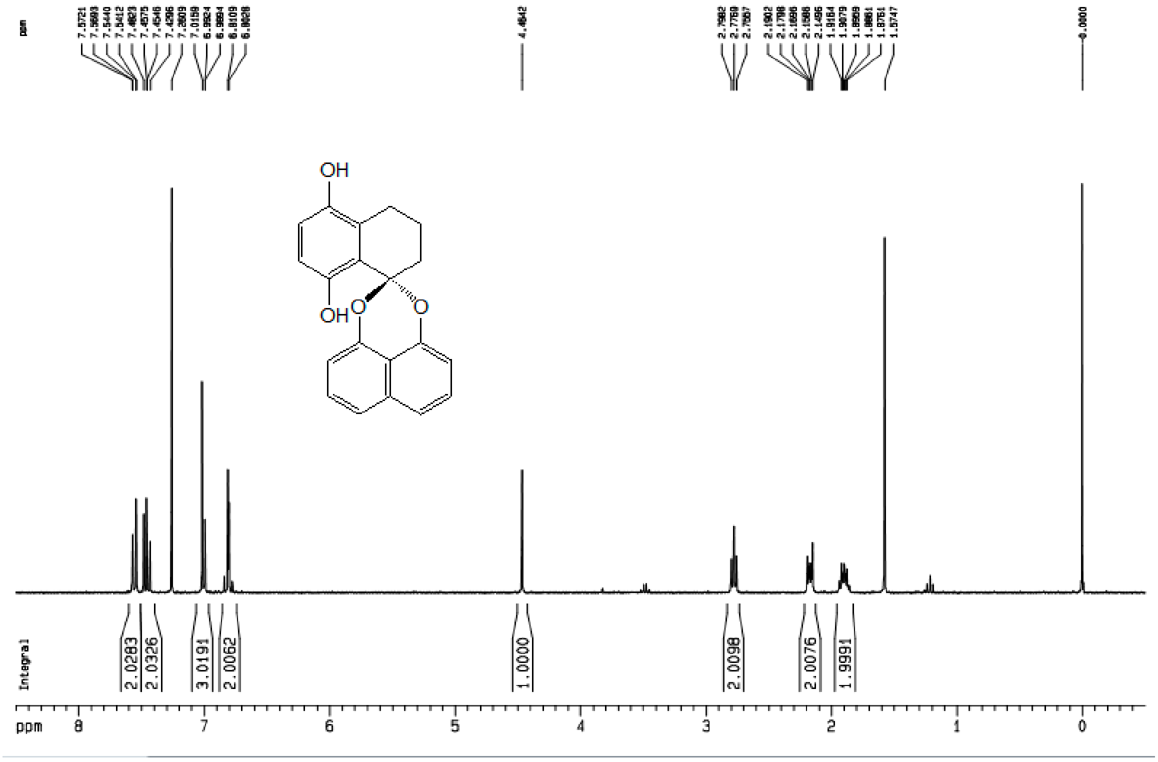

Figure S11. ^{1}H NMR of compound **9a**.

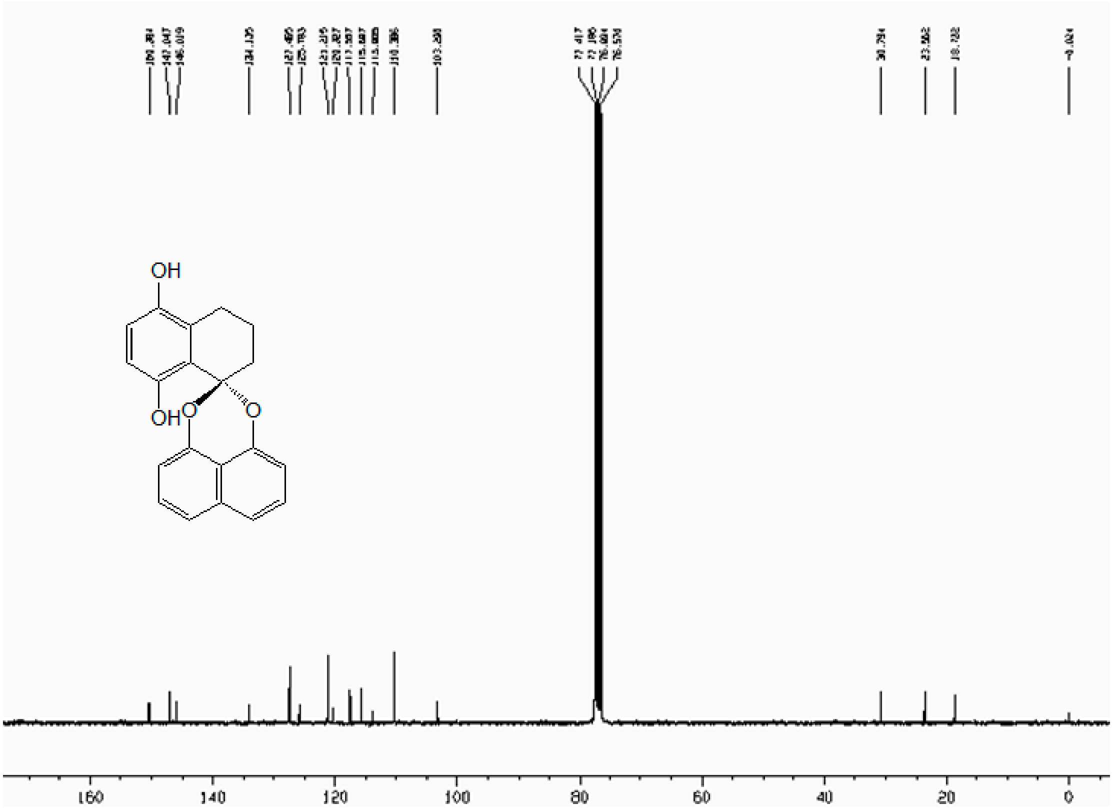

Figure S12. ^{13}C NMR of compound **9a**.

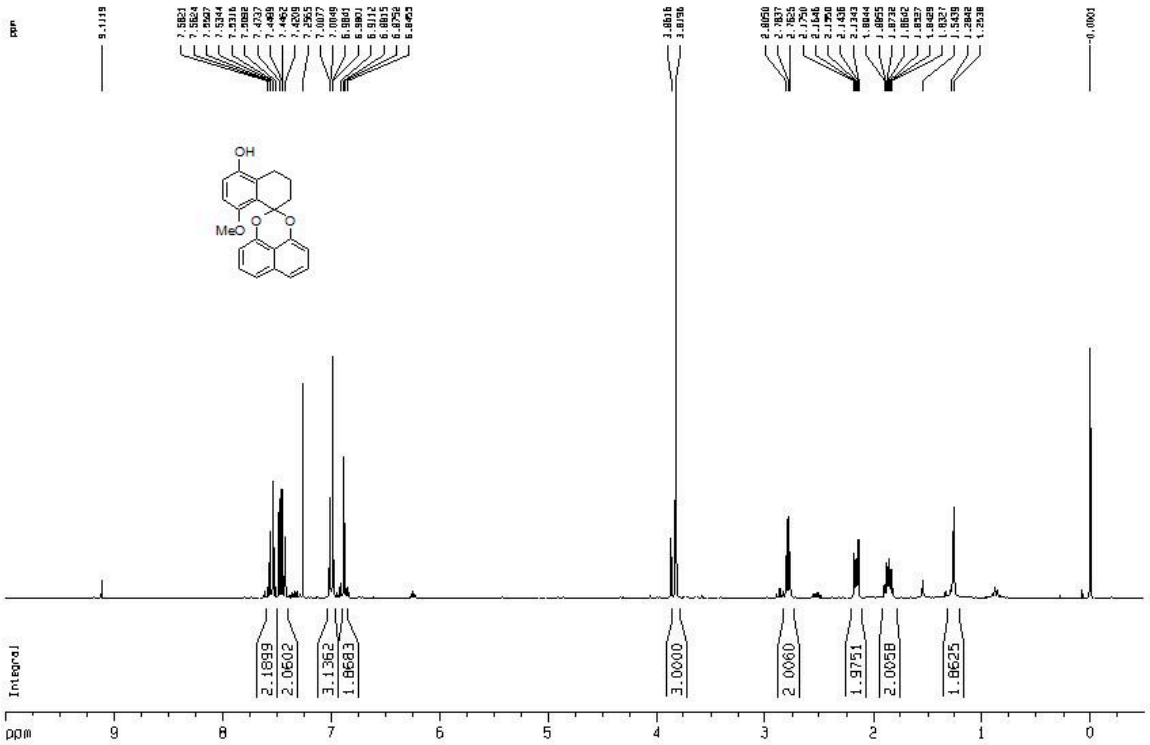

Figure S13. ^{1}H NMR of compound **9b**.

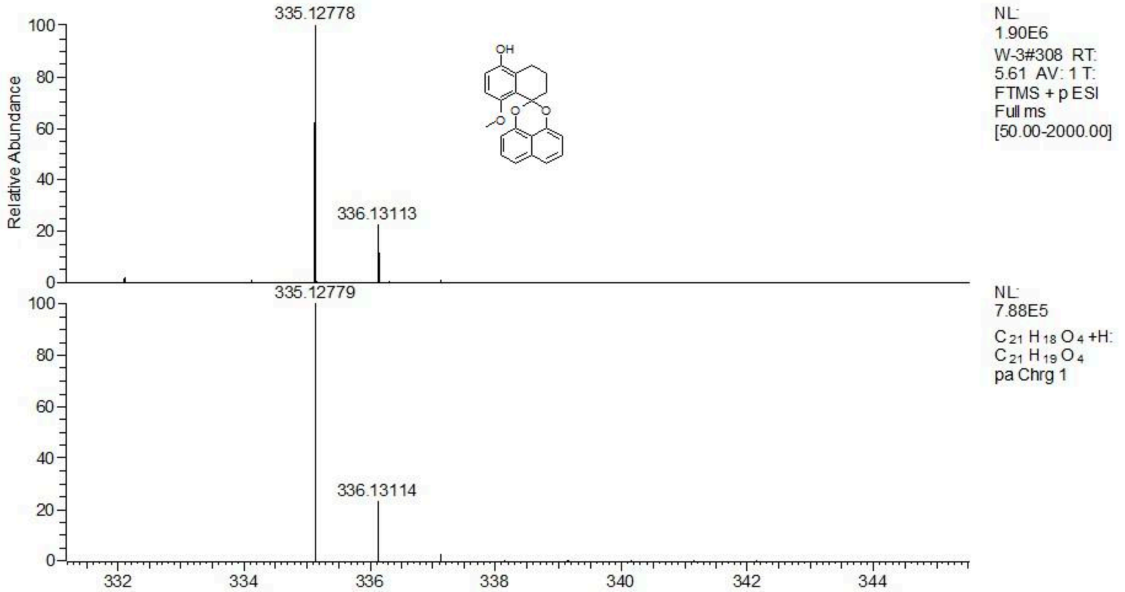

Figure S14. HR-MS (ESI) of compound **9b**.

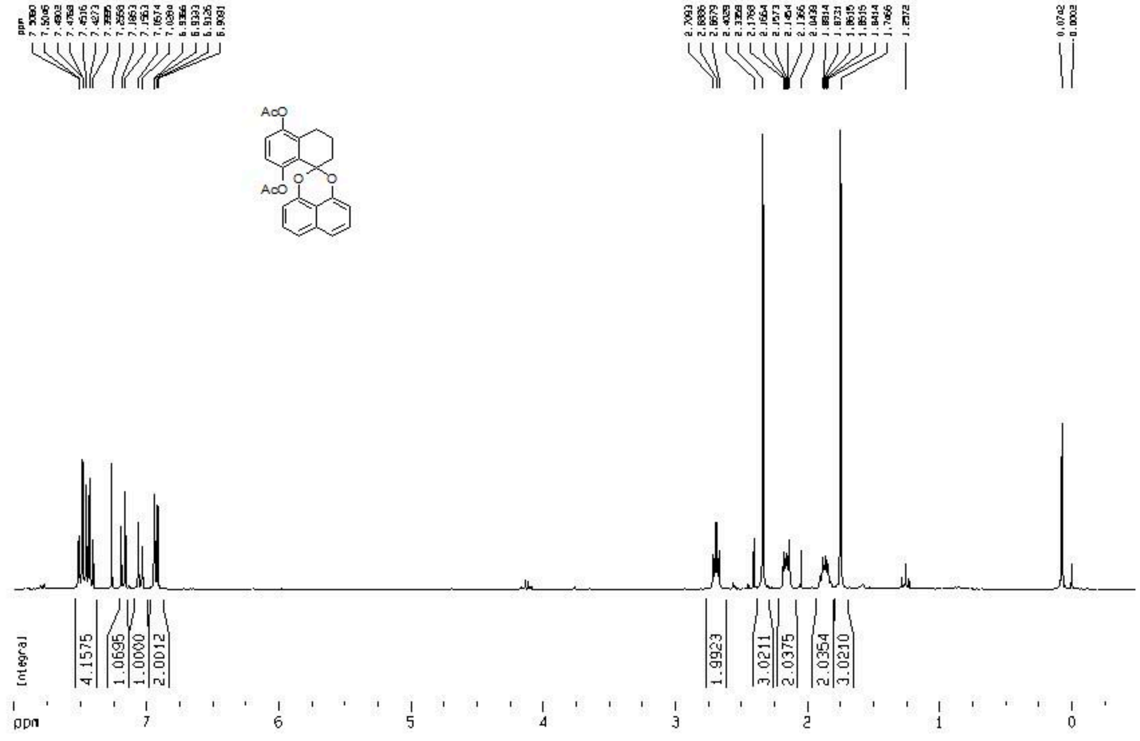

Figure S15. ^{1}H NMR of compound **10a**.

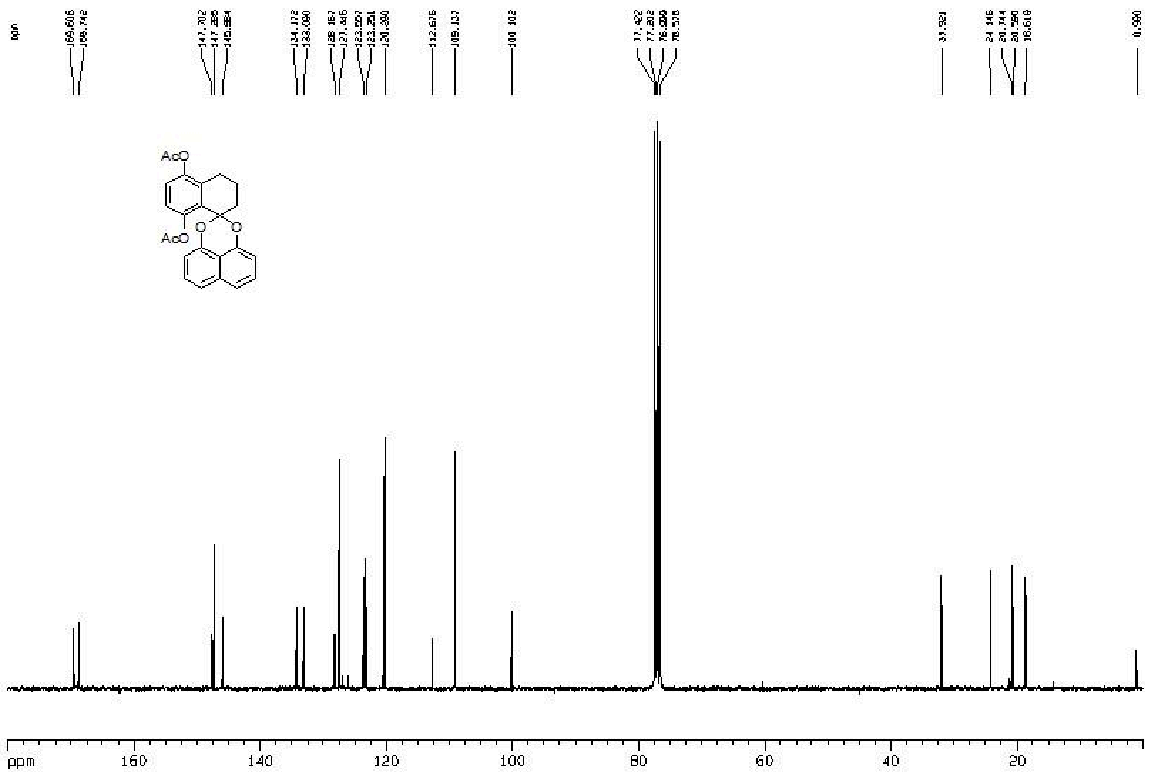

Figure S16. ^{13}C NMR of compound **10a**.

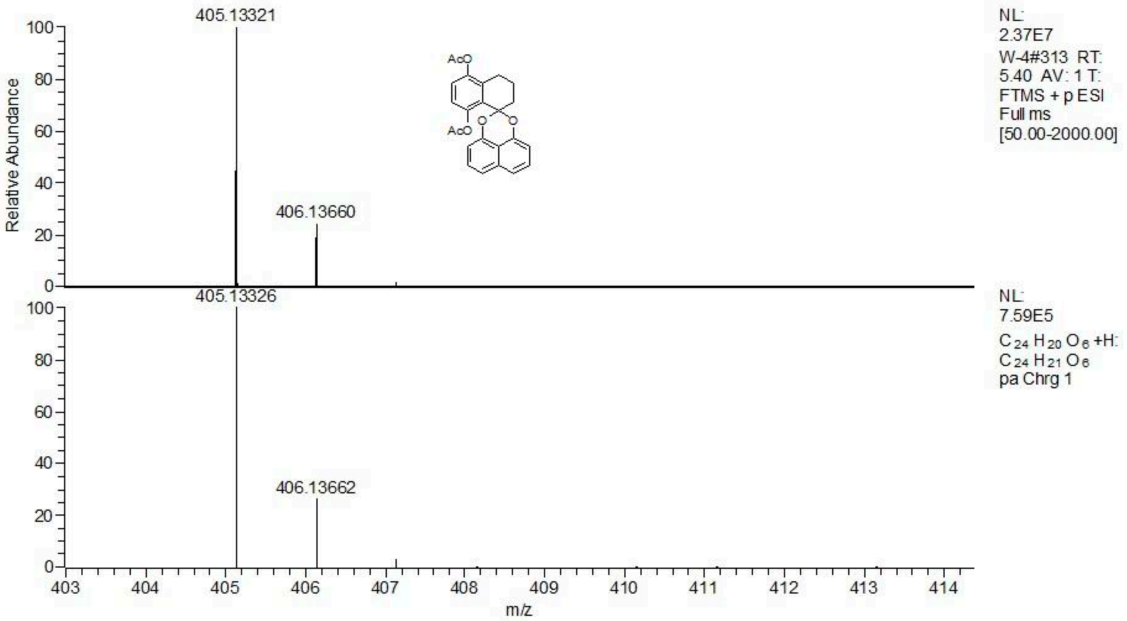

Figure S17. HR-MS (ESI) of compound **10a**.

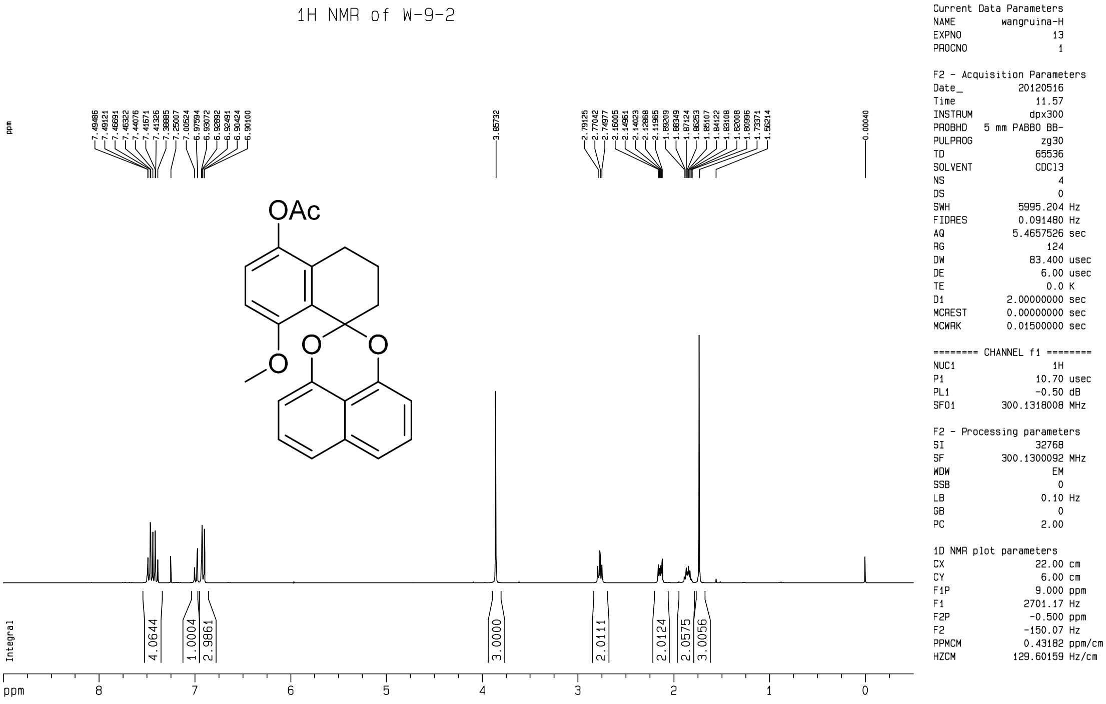

Figure S18. ^{1}H NMR of compound **10b**.

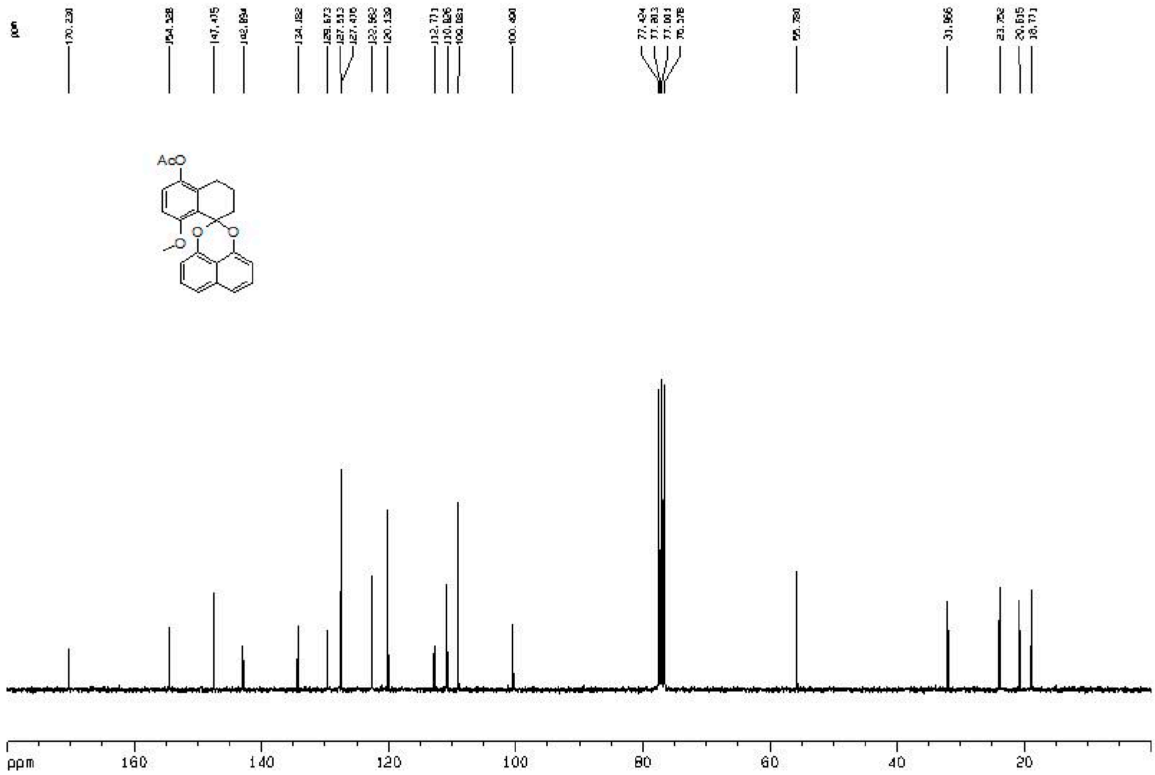

Figure S19. ^{13}C NMR of compound **10b**.

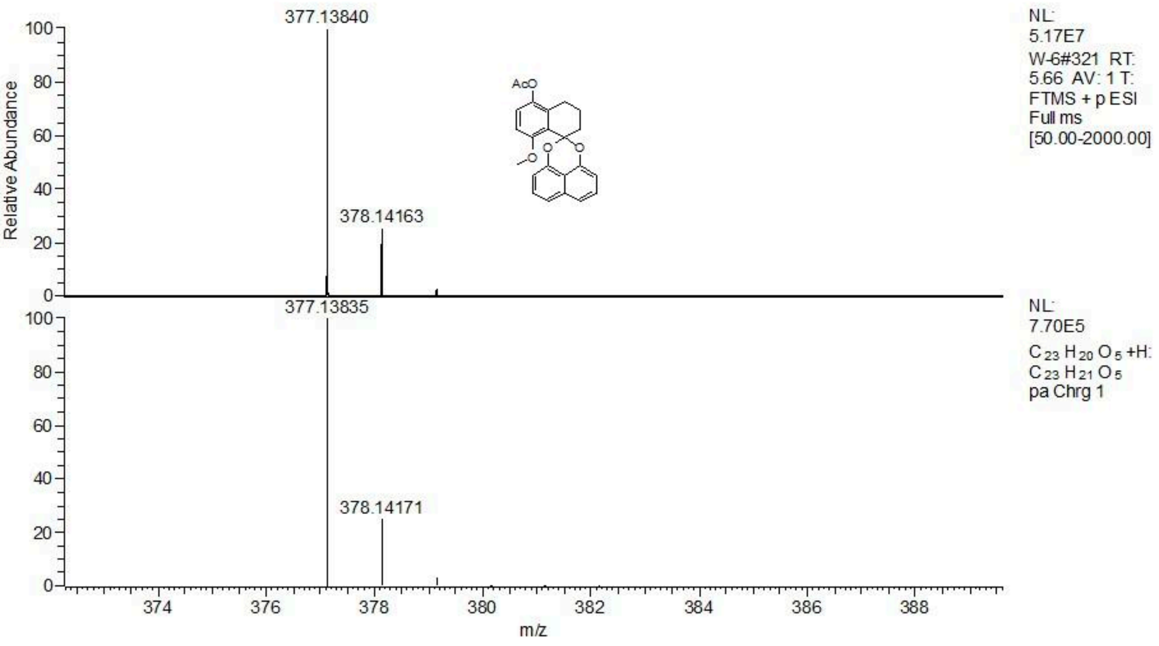

Figure S20. HR-MS (ESI) of compound **10b**.

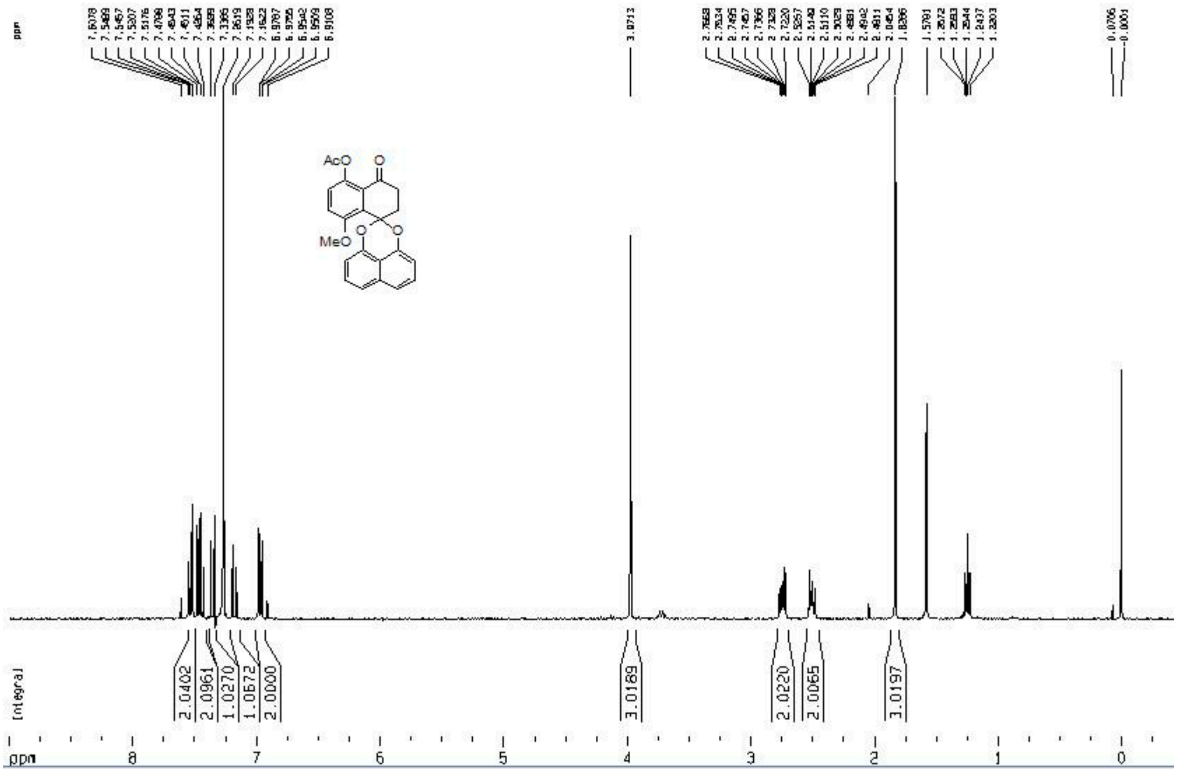

Figure S21. ^{1}H NMR of compound **11b**.

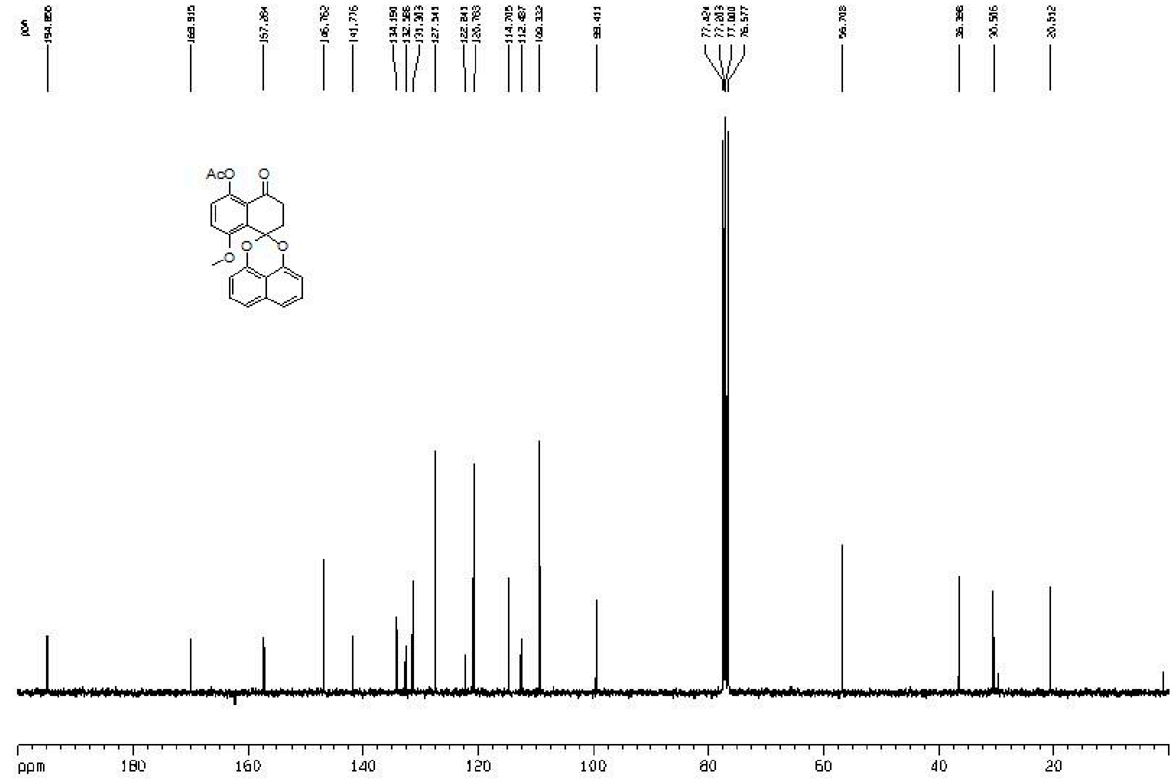

Figure S22. ^{13}C NMR of compound **11b**.

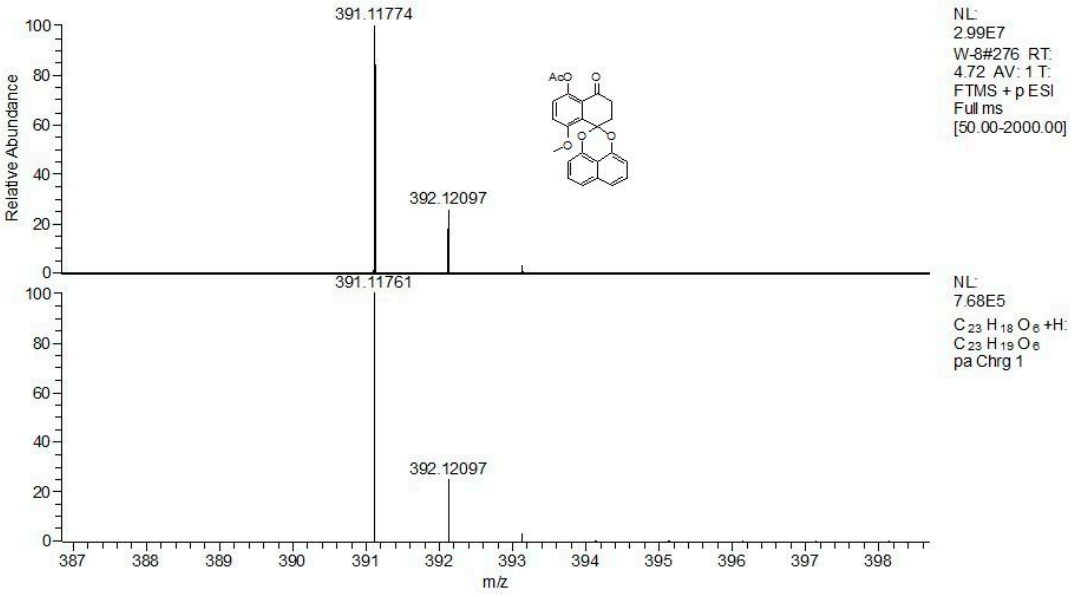

Figure S23. HR-MS (ESI) of compound **11b**.

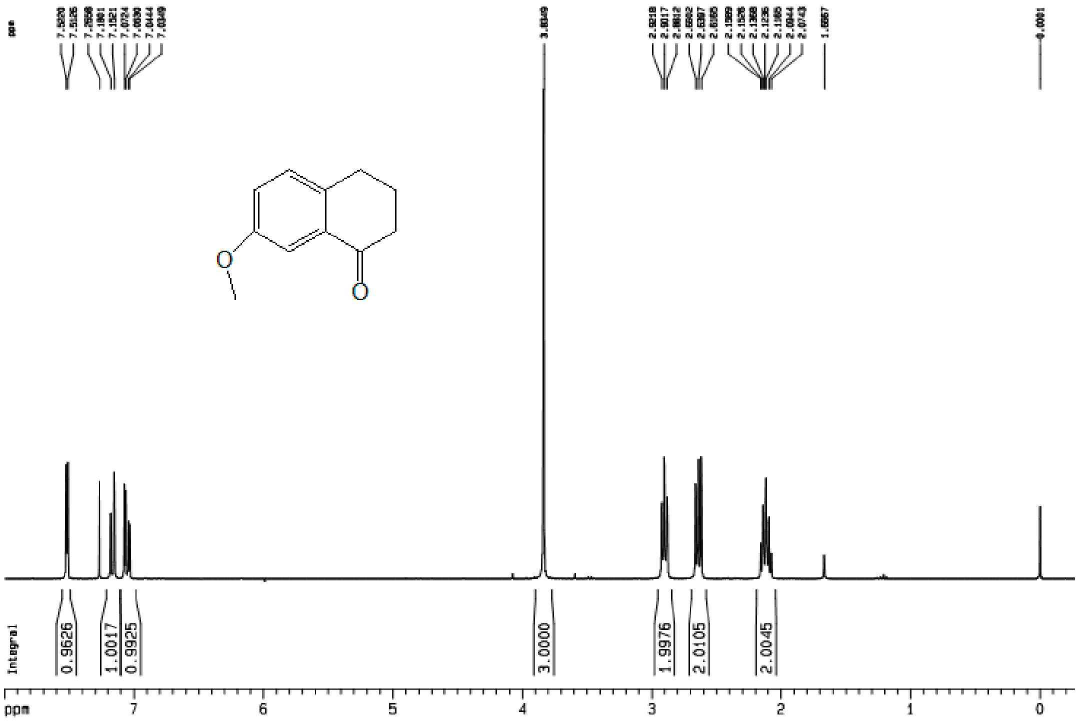

Figure S24. ^{1}H NMR of compound **13**.

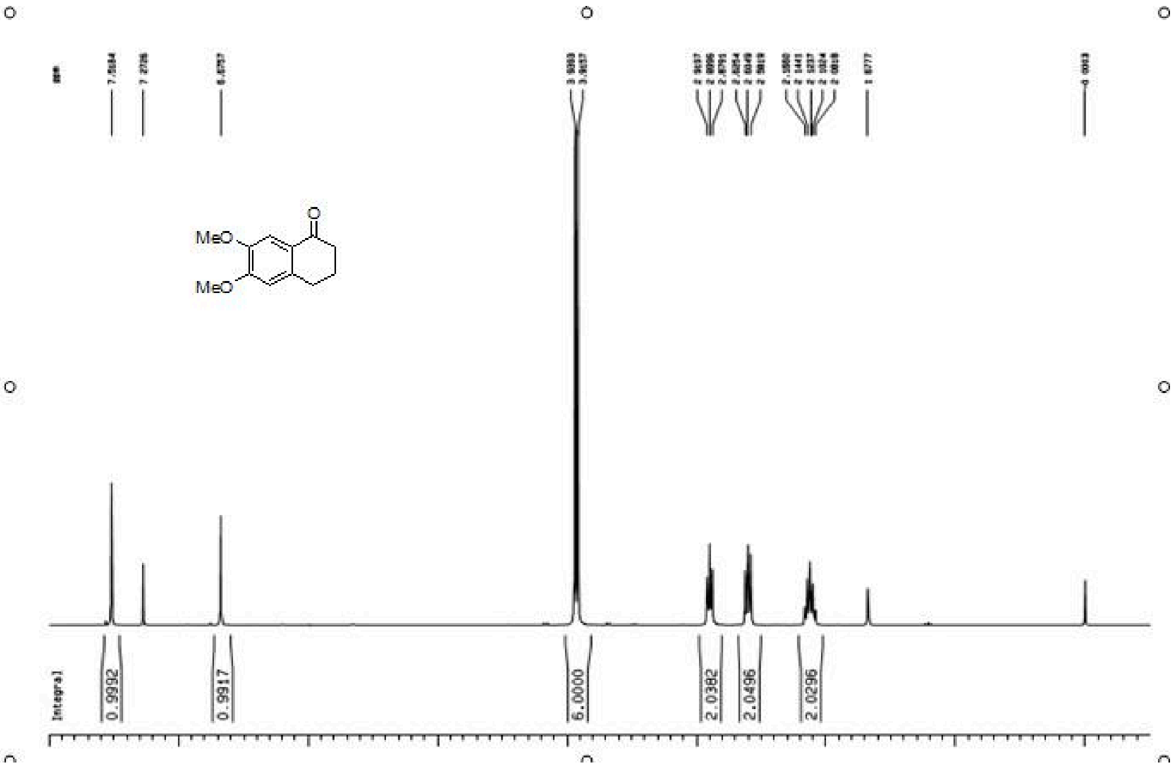

Figure S25. ^{1}H NMR of compound **14**.

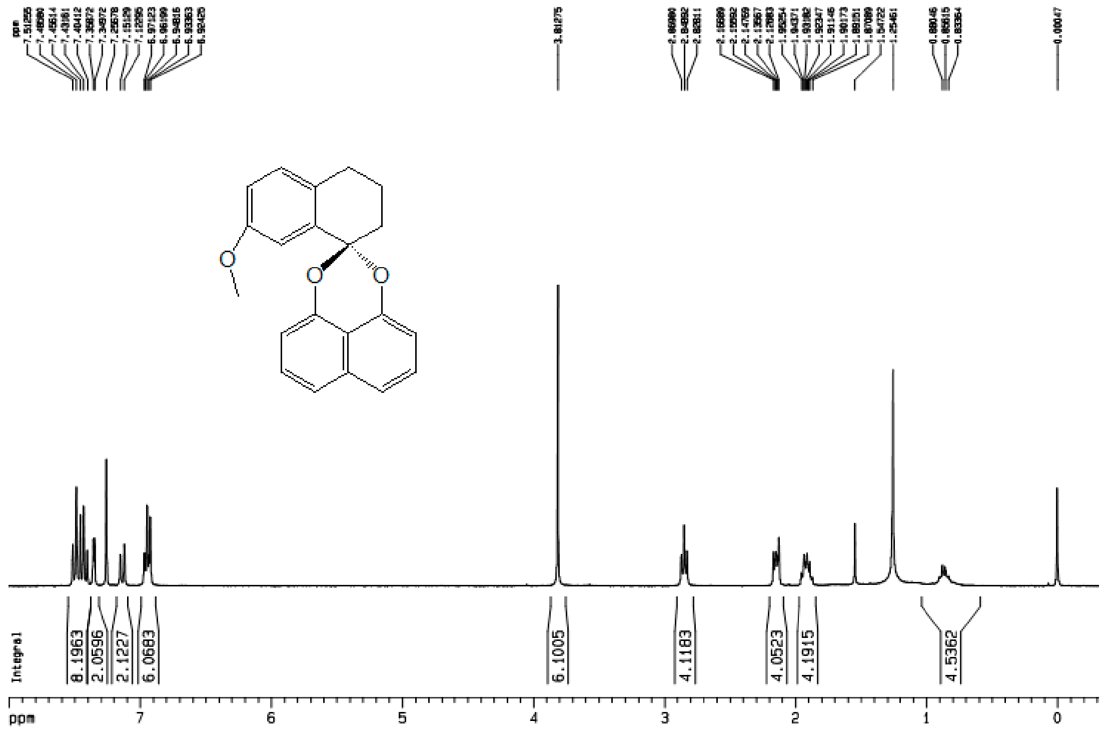

Figure S26. ^{1}H NMR of compound **15**.

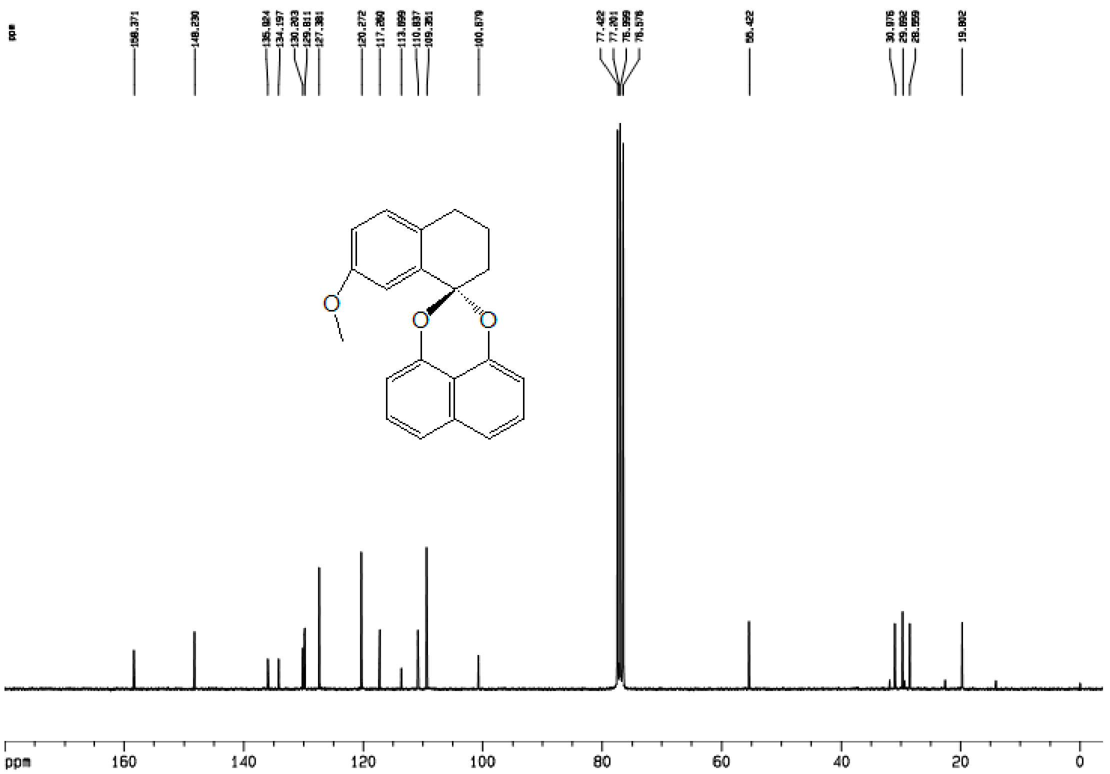

Figure S27. ^{13}C NMR of compound **15**.

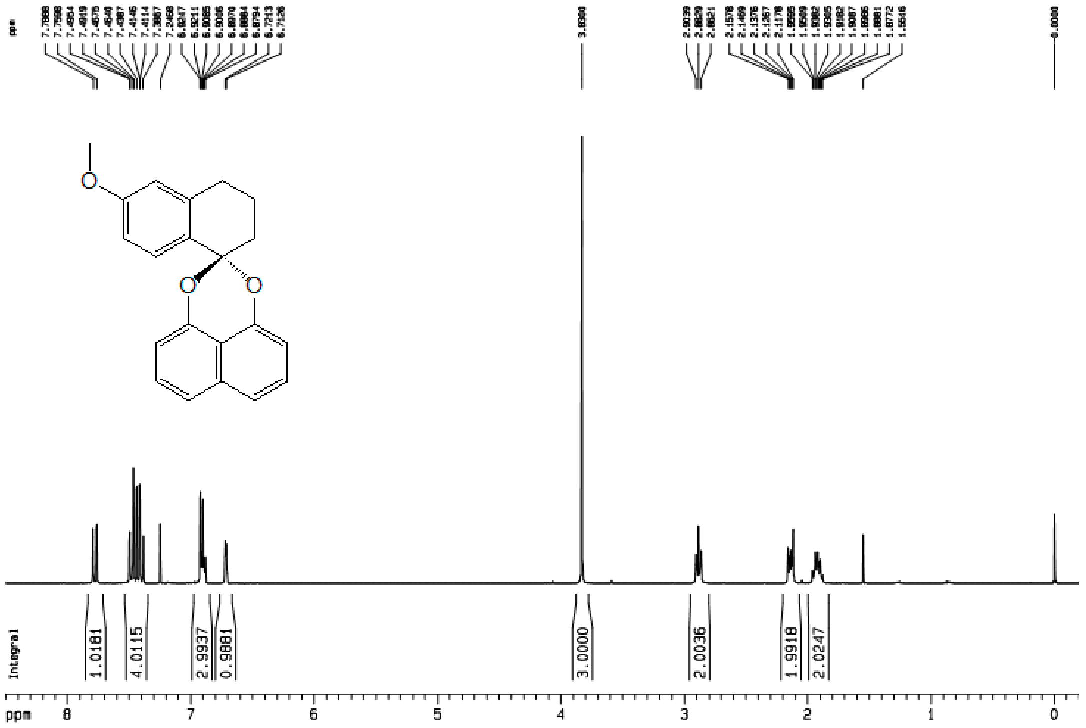

Figure S28. ^{1}H NMR of compound **16**.

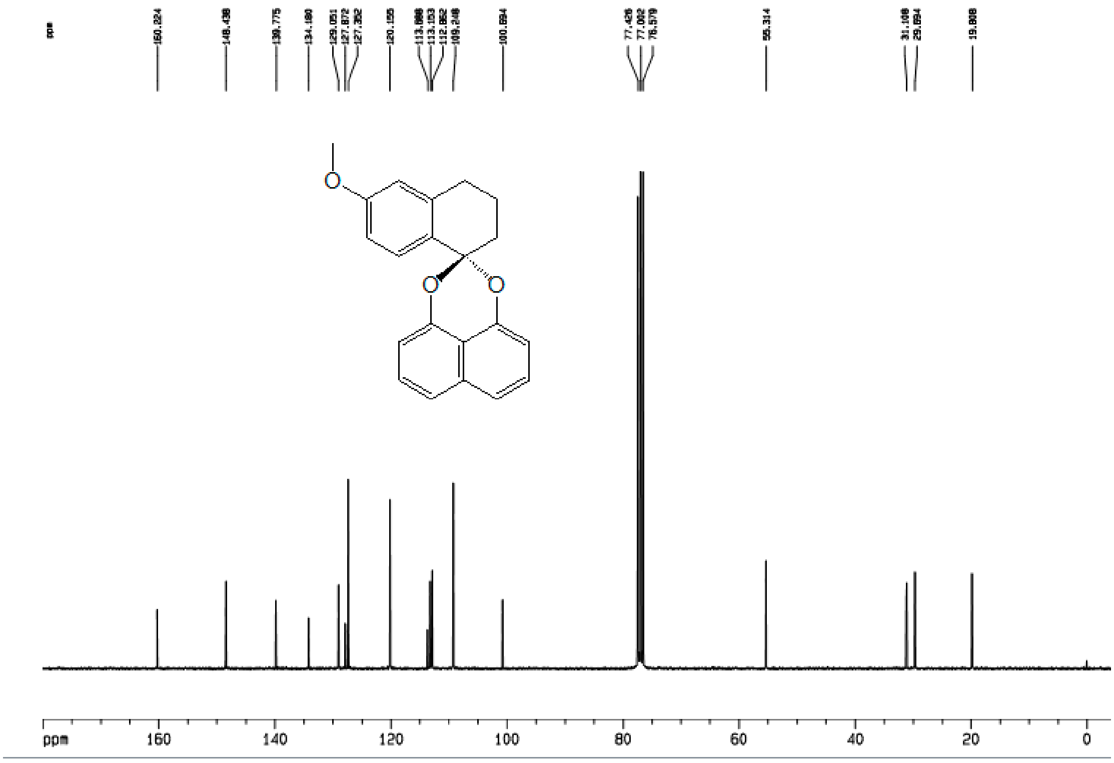

Figure S29. ^{13}C NMR of compound **16**.

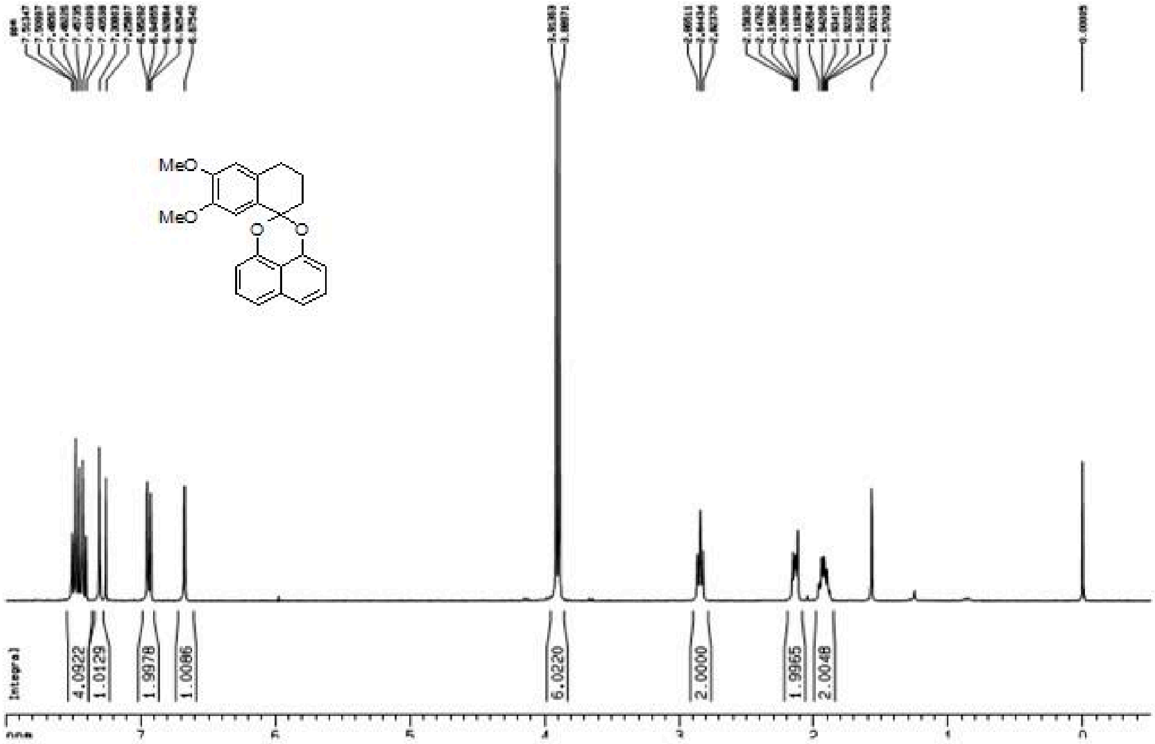

Figure S30. ^{1}H NMR of compound **17**.

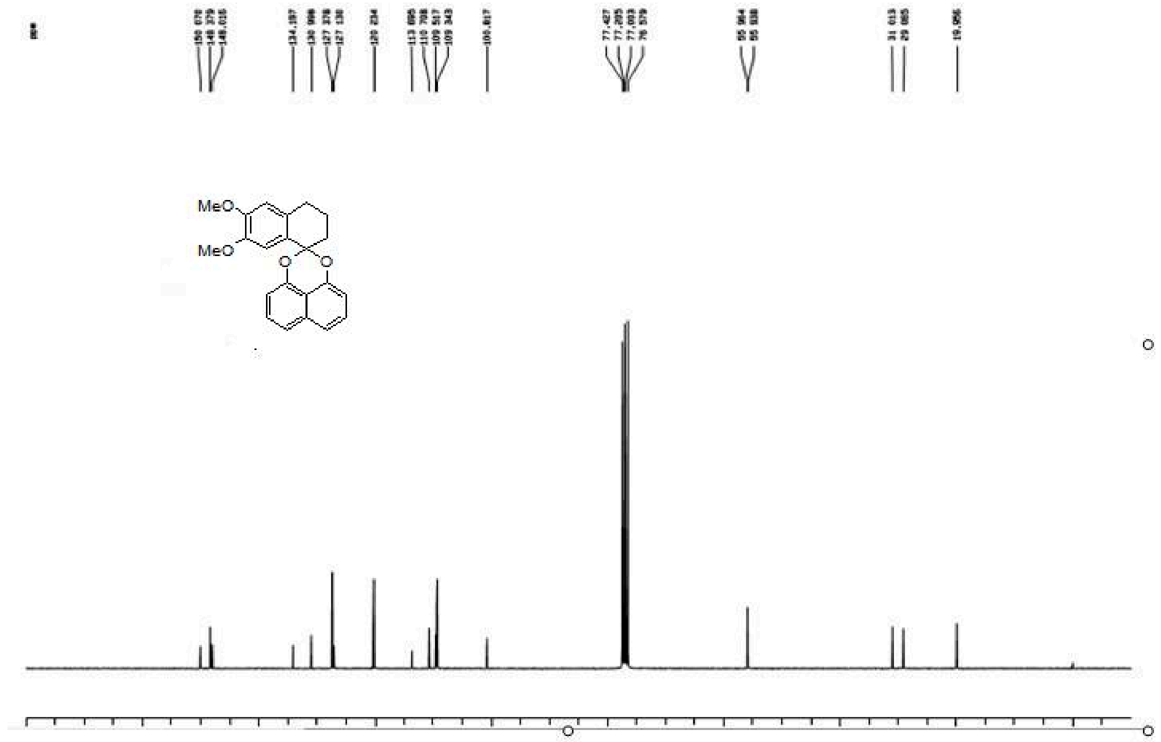

Figure S31. ^{13}C NMR of compound **17**.

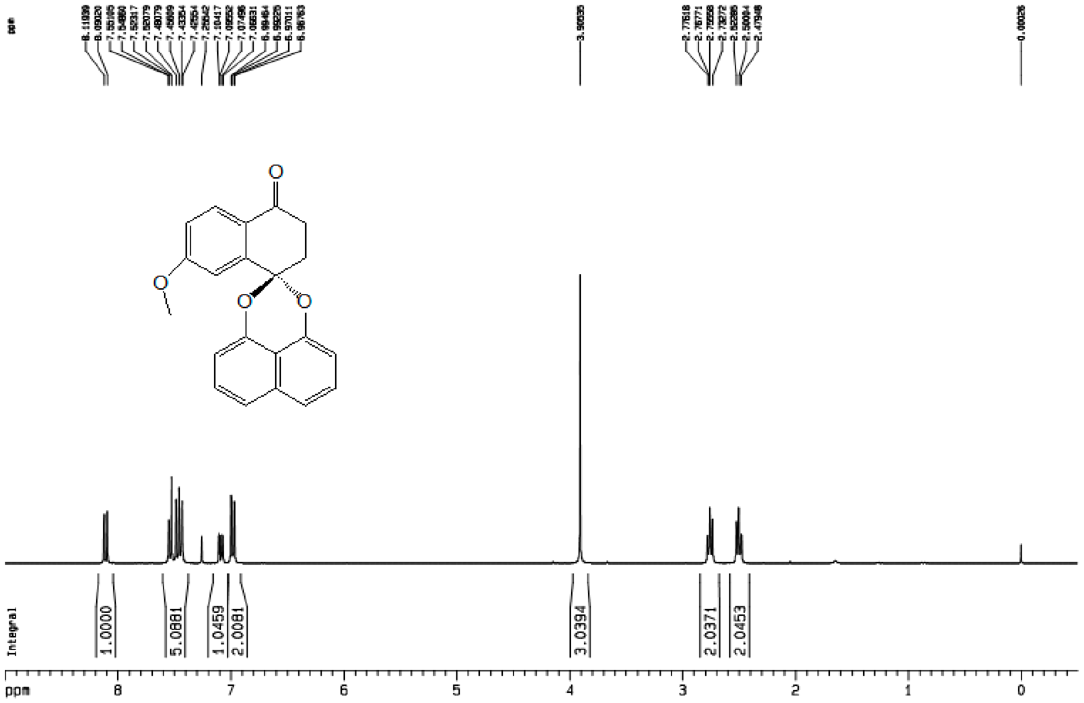

Figure S32. ^{1}H NMR of compound **18**.

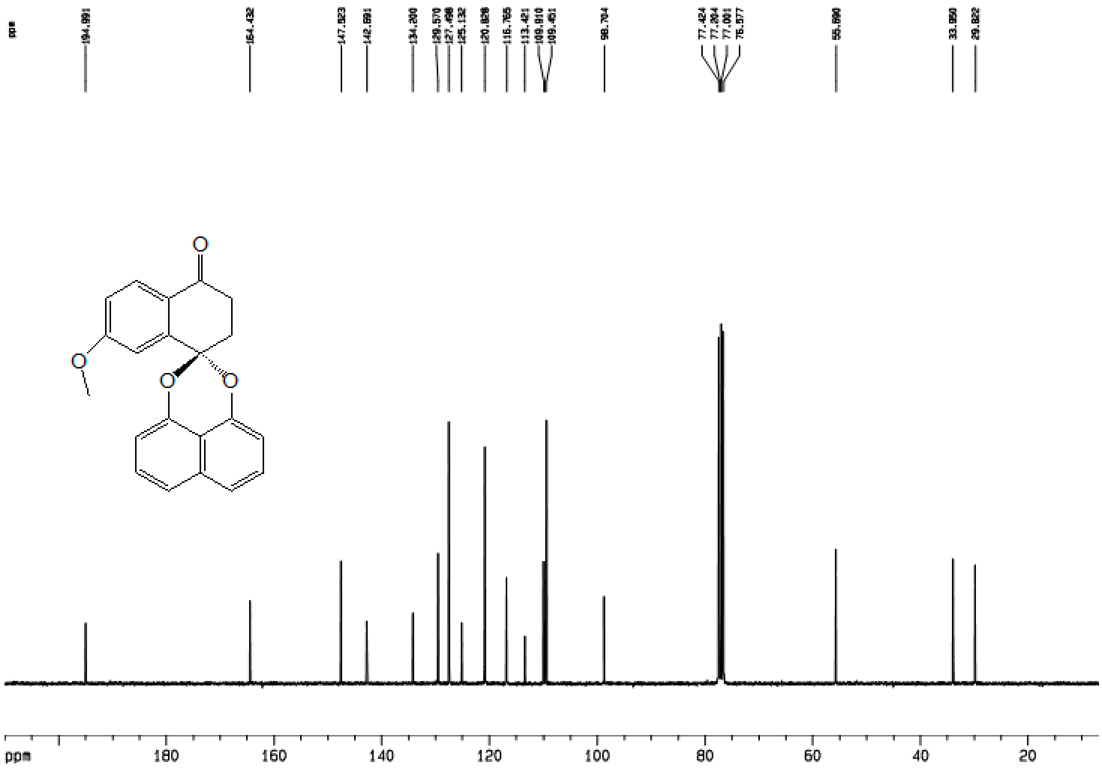

Figure S33. ^{13}C NMR of compound **18**.

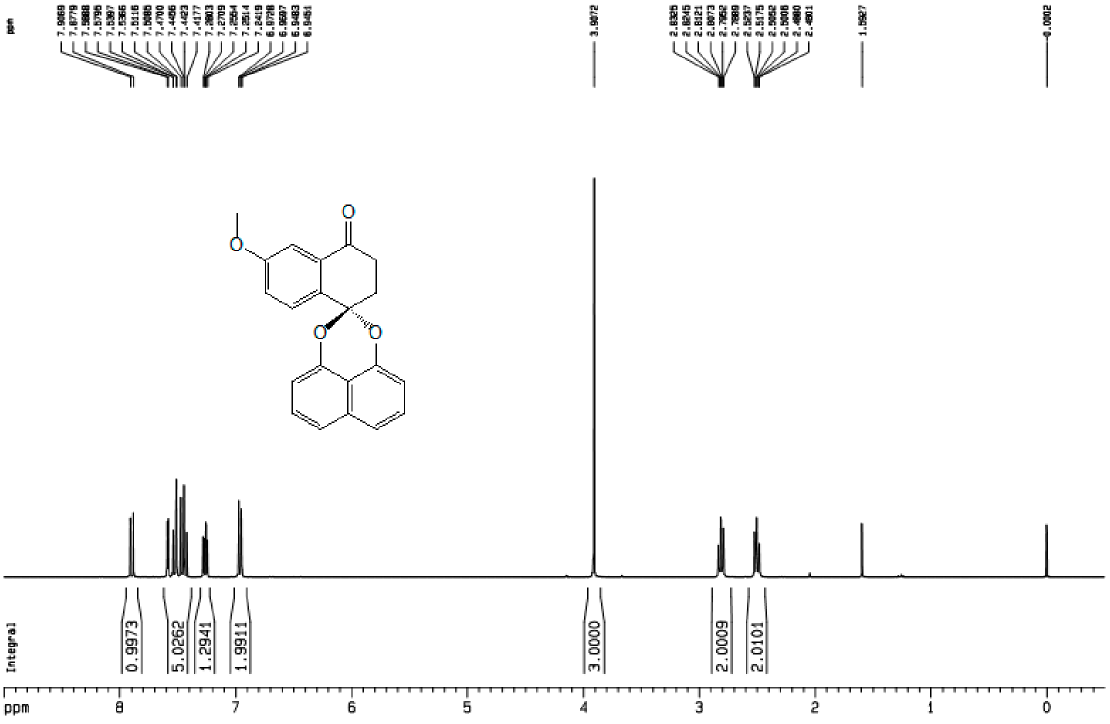

Figure S34. ^{1}H NMR of compound **19**.

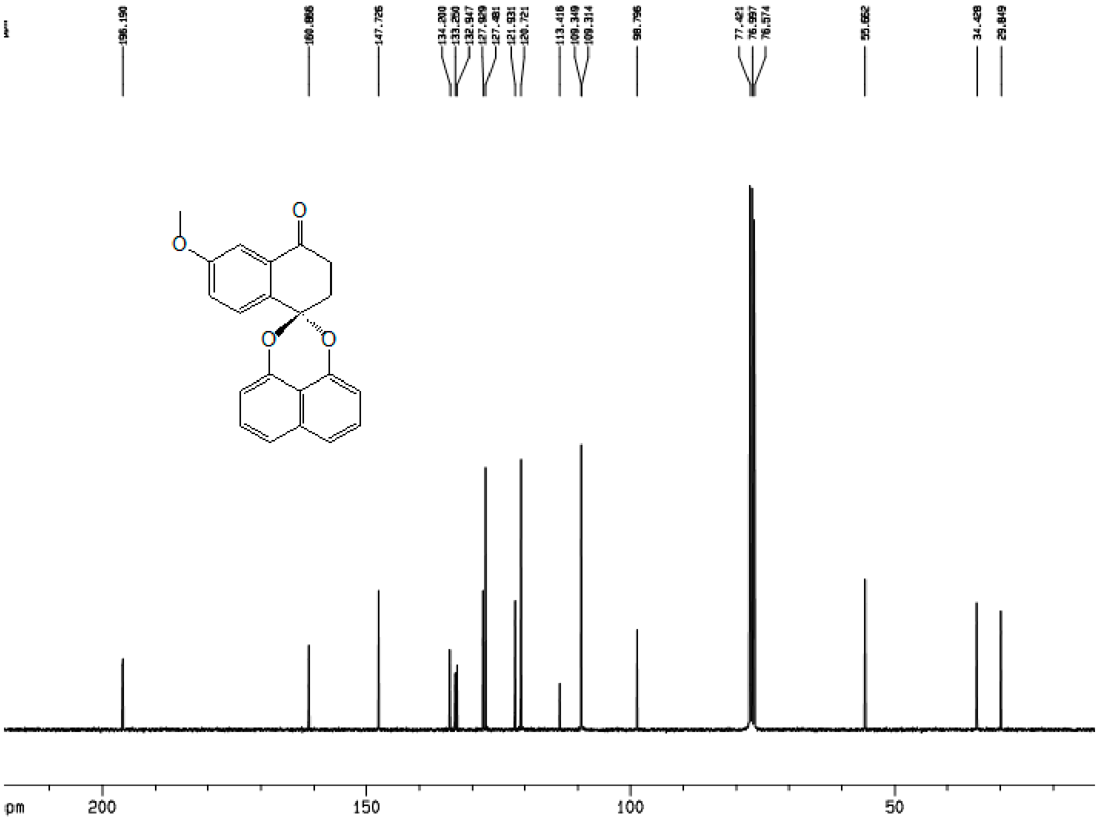

Figure S35. ^{13}C NMR of compound **19**.

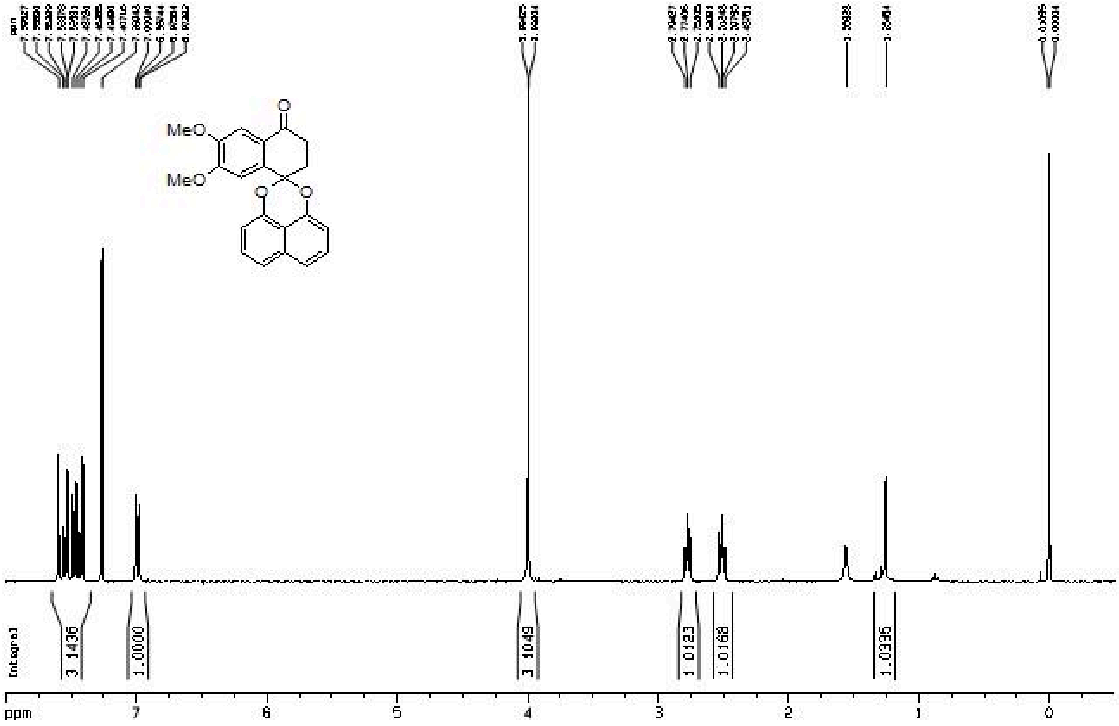

Figure S36. ^{1}H NMR of compound **20**.

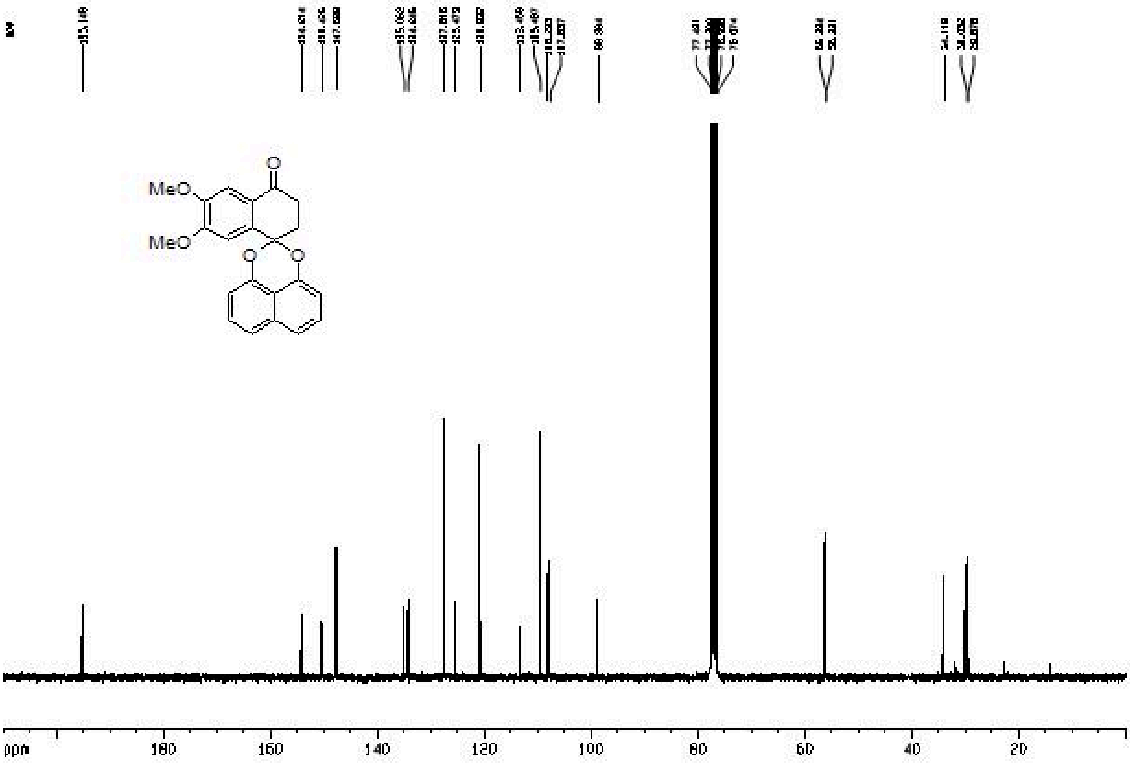

Figure S37. ^{13}C NMR of compound **20**.

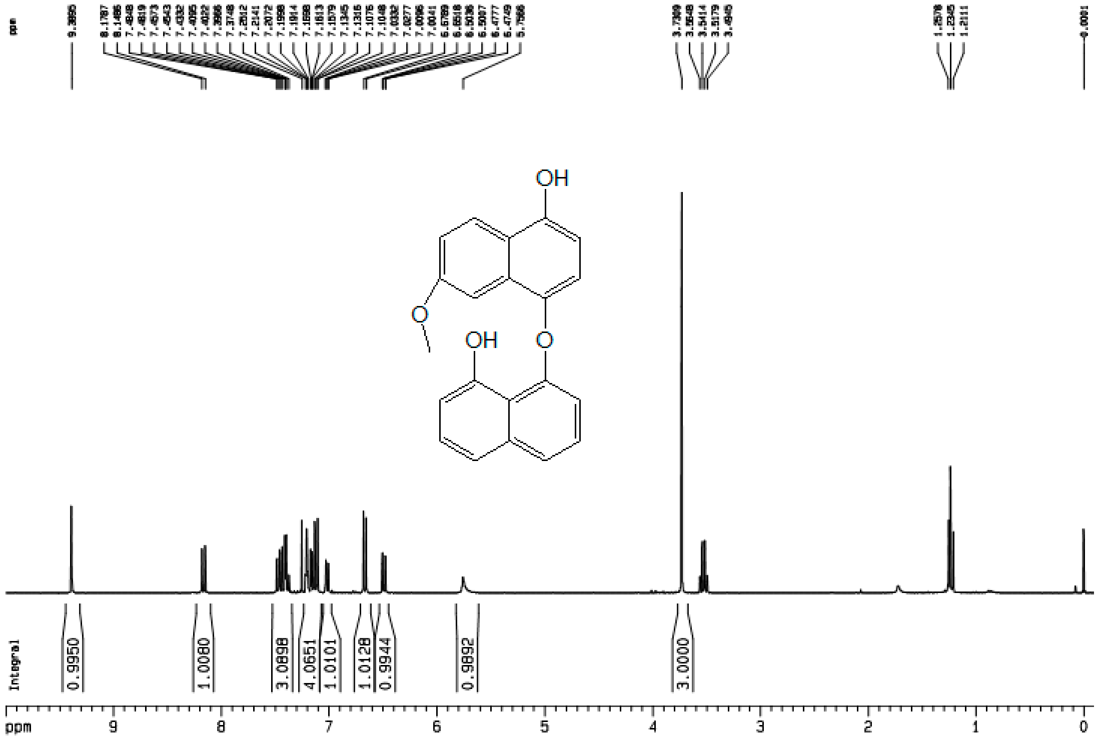

Figure S38. ^{1}H NMR of compound **21**.

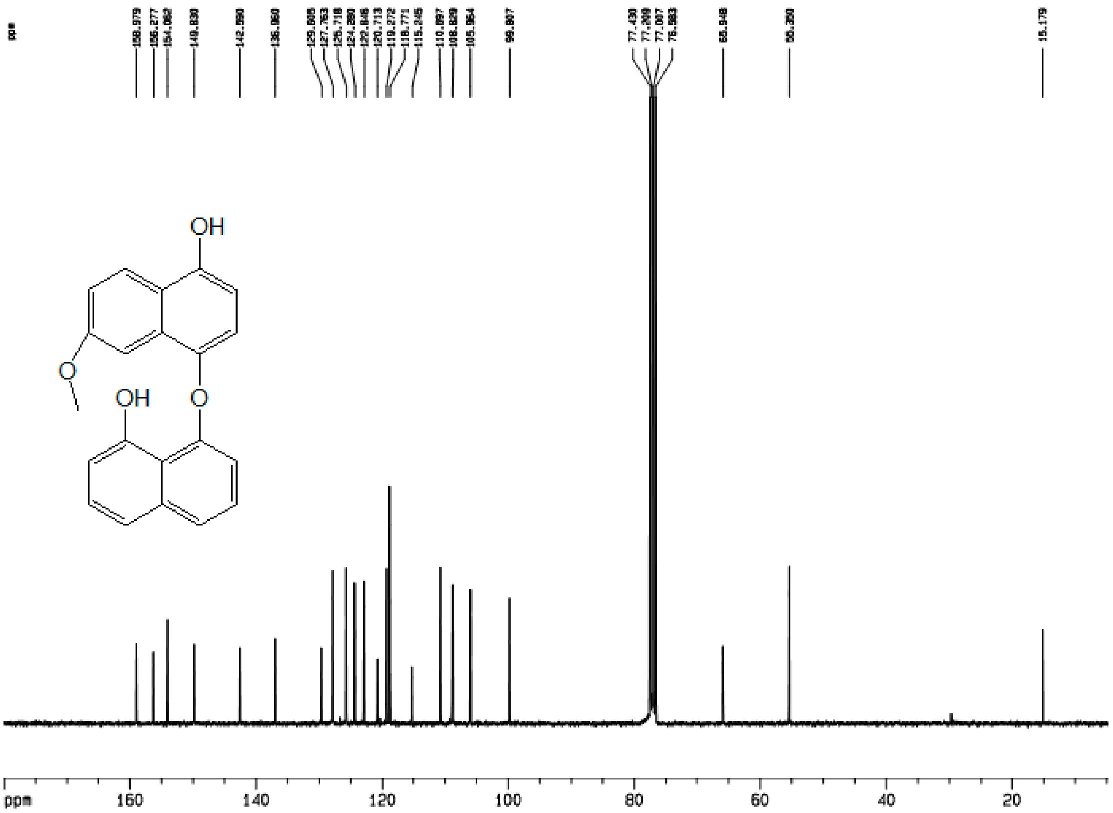

Figure S39. ^{13}C NMR of compound **21**.

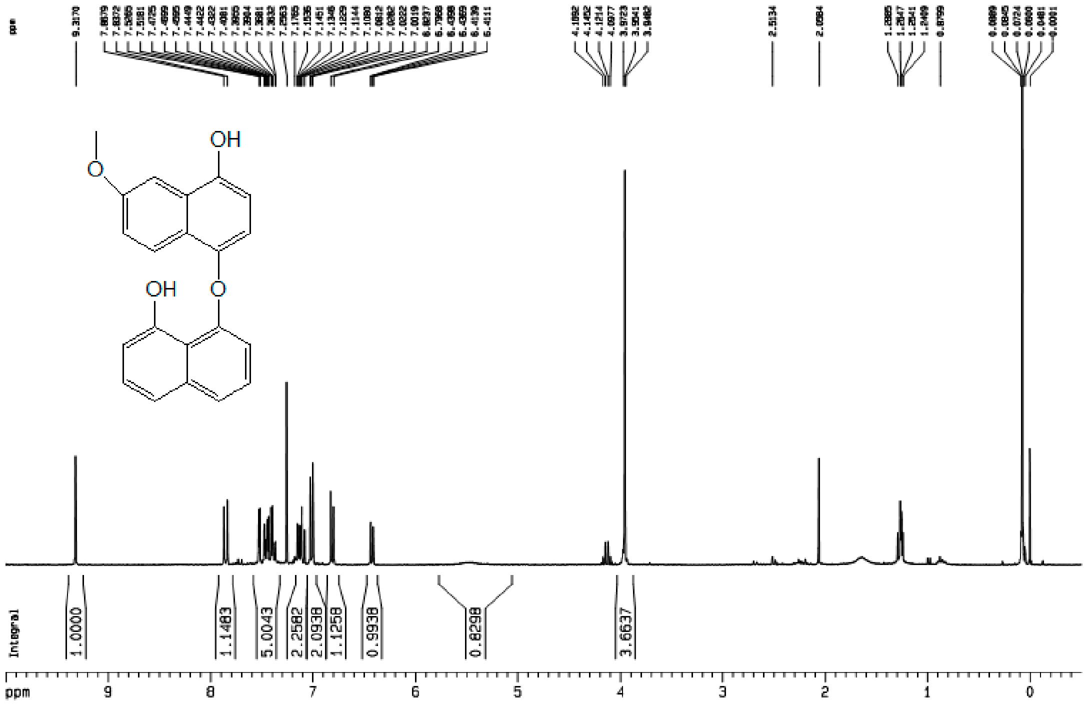

Figure S40. ^{1}H NMR of compound **22**.

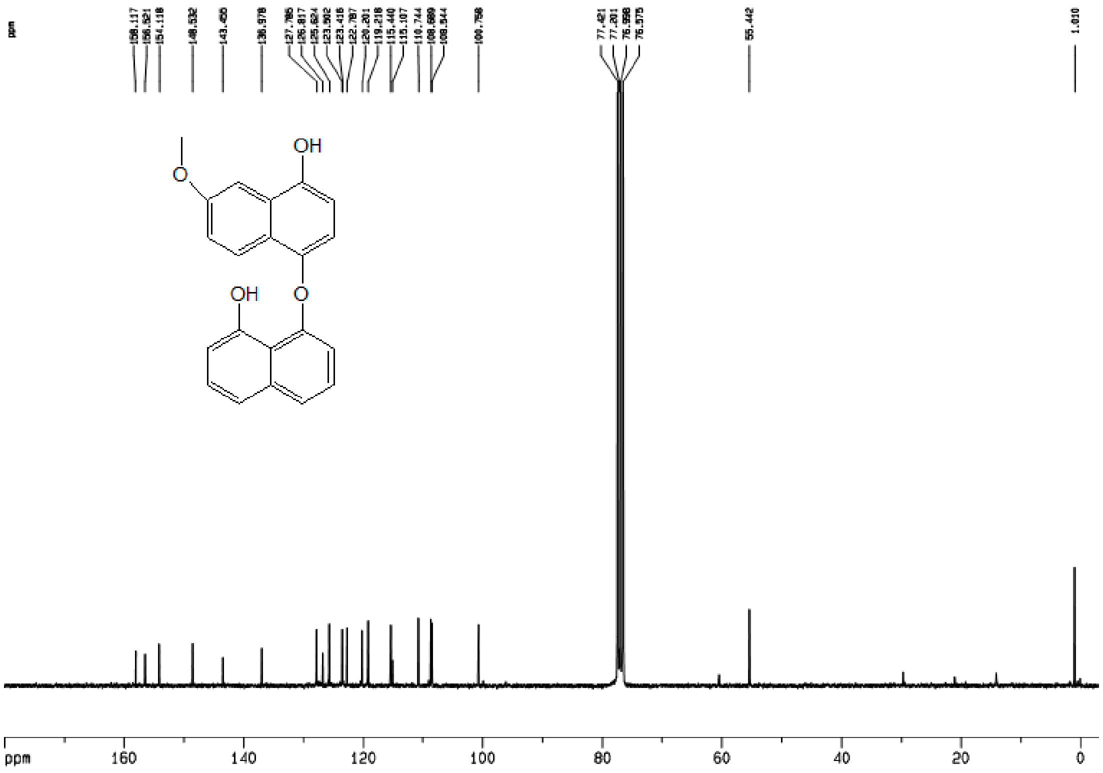

Figure S41. ^{13}C NMR of compound **22**.

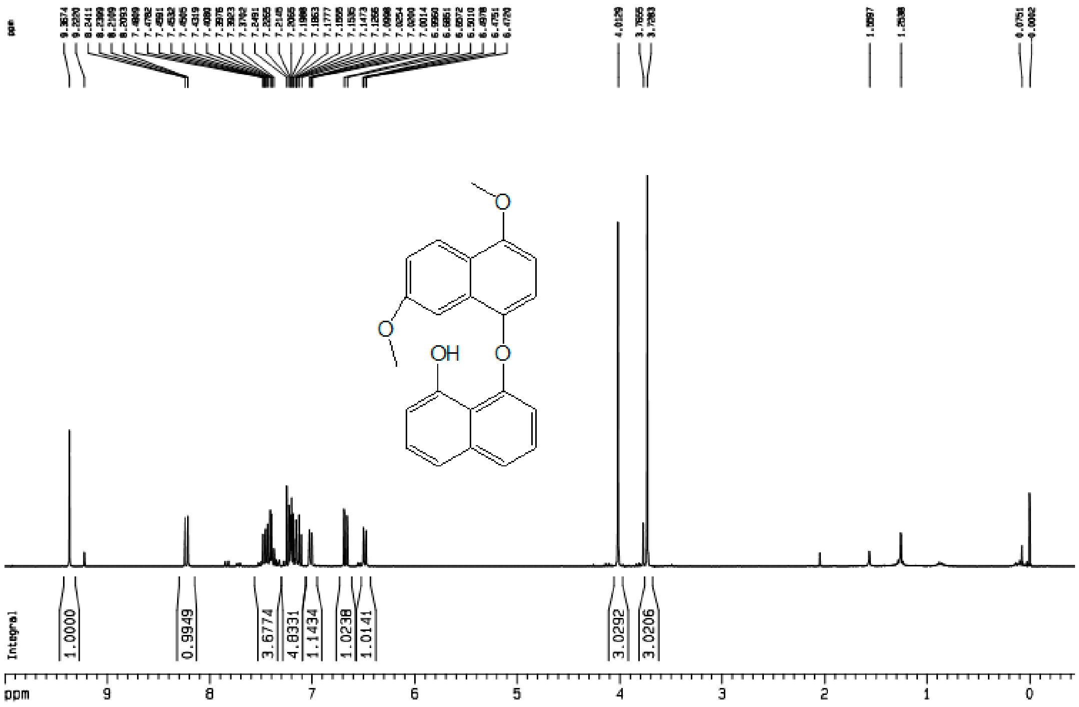

Figure S42. ^{1}H NMR of compound **23**.
